# PheCode-guided multi-modal topic modeling of electronic health records improves disease incidence prediction and GWAS discovery from UK Biobank

**DOI:** 10.1093/bib/bbag030

**Published:** 2026-02-02

**Authors:** Ziqi Yang, Ziyang Song, Shadi Zabad, Marc-André Legault, Yue Li

**Affiliations:** School of Computer Science, McGill University, 3480 Rue University, Montréal, QC, H3A 2A7, Canada; School of Computer Science, McGill University, 3480 Rue University, Montréal, QC, H3A 2A7, Canada; School of Electrical Engineering and Computer Science, Ohio University, 1 Ohio University, Athens, OH, 45701, United States; School of Computer Science, McGill University, 3480 Rue University, Montréal, QC, H3A 2A7, Canada; Faculty of Pharmacy, Université de Montréal, 2940 Chem. de Polytechnique, Montréal, QC, H3T 1J4, Canada; School of Computer Science, McGill University, 3480 Rue University, Montréal, QC, H3A 2A7, Canada

**Keywords:** electronic health records, topic modeling, phenotyping, genome-wide association study, disease incidence prediction, machine learning

## Abstract

Phenome-wide association studies rely on disease definitions derived from diagnostic codes, often failing to leverage the full richness of electronic health records (EHR). We present MixEHR-SAGE, a PheCode-guided multi-modal topic model that integrates diagnoses, procedures, and medications to enhance phenotyping from large-scale EHRs. By combining expert-informed priors with probabilistic inference, MixEHR-SAGE identifies over 1000 interpretable phenotype topics from UK Biobank data. Applied to 350 000 individuals with high-quality genetic data, MixEHR-SAGE-derived risk scores accurately predict incident type 2 diabetes (T2D) and leukemia diagnoses. Subsequent genome-wide association studies using these continuous risk scores uncovered novel disease-associated loci, including *PPP1R15A* for T2D and *JMJD6*/*SRSF2* for leukemia, that were missed by traditional binary case definitions. These results highlight the potential of probabilistic phenotyping from multi-modal EHRs to improve genetic discovery. The MixEHR-SAGE software is publicly available at: https://github.com/li-lab-mcgill/MixEHR-SAGE.

## Introduction

Phenome-wide association studies (PheWAS) are widely used to identify associations between genetic variants and a diverse set of diseases [1, 2]. These studies are enabled by the growing availability of genetic and electronic health record (EHR) data in large cohorts, such as the UK Biobank (UKB). The UKB includes over 500 000 participants with available genotyping data, self-reported pharmacotherapy data and linkage to various health records and registries providing hospitalization diagnostic codes and surgical procedure codes. The extensive phenotypic characterization of UKB participants, in combination with the availability of genetic data, has enabled large-scale PheWAS with results in online repositories (e.g. OpenTargets [3]).

However, medico-administrative records represent an imperfect view of participant’s health, representing a challenge in PheWAS [4]. In PheWAS, researchers commonly rely on diagnostic algorithms that combine International Classification of Diseases (ICD) 9 and 10 codes to define disease status for genetic association testing [5–7]. The most commonly used mapping of ICD codes used in PheWAS are called *Phenotype Codes (PheCodes)* [8]. These PheCodes were defined manually and are organized in categories (e.g. cardiovascular system, digestive, neoplasms, etc.), and span most health-related phenotypes. An important limitation of PheCodes is that they only rely on ICD codes and do not leverage complementary sources of information, such as pharmacotherapy data or surgical procedure codes. Furthermore, the validity of PheCodes may vary between studies and datasets due to differences in ICD code usage between physicians or institutions, motivating the development of adaptive systems.

Topic models offer a powerful and data-driven approach to create algorithmic disease definitions. They were developed to summarize documents based on a mixture of latent topics defined by statistical word co-occurrences in the field of natural language processing [9, 10]. By analogy, EHRs can be viewed as “documents,” where co-occurring diagnostic codes capture the unobserved disease phenotypes, and patient-level topic mixtures can be interpreted as continuous disease scores. MixEHR uses topic models to infer a joint distribution across various EHR data types—such as diagnostic codes, self-reported pharmacotherapy data, and surgical procedure codes—to more comprehensively capture disease status from complex EHR data [11]. Usually, the use of topic models is hampered by the need for the *post hoc* interpretation of latent disease topics, as they are learned without disease labels. This characteristic is shared with other latent variable models, such as principal component (PC) analysis and non-negative matrix factorization which are frequently used to analyze the high-dimensional EHR data and uncover hidden disease representations in the context of phenotyping and risk stratification [12]. Recent advances in topic models developed for EHR data alleviate this problem [13, 14]. Namely, MixEHR-G incorporates expert-defined phenotype mappings to initialize topic priors, assigning higher weights to phenotype topics with respect to a patient’s observed PheCodes; MixEHR-seed infers expert-guided phenotype representations with a mixture of “seed” topics and regular topics, where a seed is referred to as clinically related ICD codes for a given PheCode. Overall, MixEHR-G employs an expert-guided prior that confines the inferred phenotype topics to predefined PheCodes. In contrast, MixEHR-Seed relies solely on expert-guided topic inference, but may face convergence issues due to uninformative initialization. Thus, to leverage the strengths of both approaches, we seek to develop a novel approach that integrates expert knowledge into initialization and adaptively controls expert-guided topic inference through learned weights.

In this study, we present a PheCode-guided, multi-modal topic model called MixEHR-SAGE (Seed-and-Guided). Our approach uses existing disease codings from PheCodes to infer 1213 phenotype topics, which can leverage complementary information from other EHR data types in a data-driven manner. Specifically, MixEHR-SAGE uses PheCodes to initialize topic priors and infers seed-guided phenotype topic distributions. While MixEHR-SAGE uses PheCodes to guide the topic inference for the ICD codes, unguided modalities such as medication use and medical procedures, which are not captured in ICD codes, still benefit from this information without requiring prespecified disease definitions. We evaluate MixEHR-SAGE on large-scale EHR datasets, including the UKB and MIMIC-III intensive care unit datasets. Our analyses demonstrate that MixEHR-SAGE identifies meaningful disease topics, enhances disease prediction, and discovers novel genetic associations.

MixEHR-SAGE integrates two complementary advances from our prior work into a unified framework for genome-wide association studies (GWAS) analysis. First, it leverages PheCode-driven prior initialization from MixEHR-G [13] to initialize disease topics. Second, it extends the seed-guided topic inference of MixEHR-Seed [14] to learn multi-modal topics anchored by PheCodes over diagnoses, procedures, and medications. Moreover, it outputs continuous disease-specific phenotype scores that can be used directly as quantitative traits for incidence prediction and GWAS. MixEHR-SAGE also scales efficiently to large-scale EHR datasets such as UKB, enabled by its variational inference algorithm and ability to learn from sparse multi-modal inputs.

## Materials and methods

### MixEHR-SAGE overview

**Figure 1:**
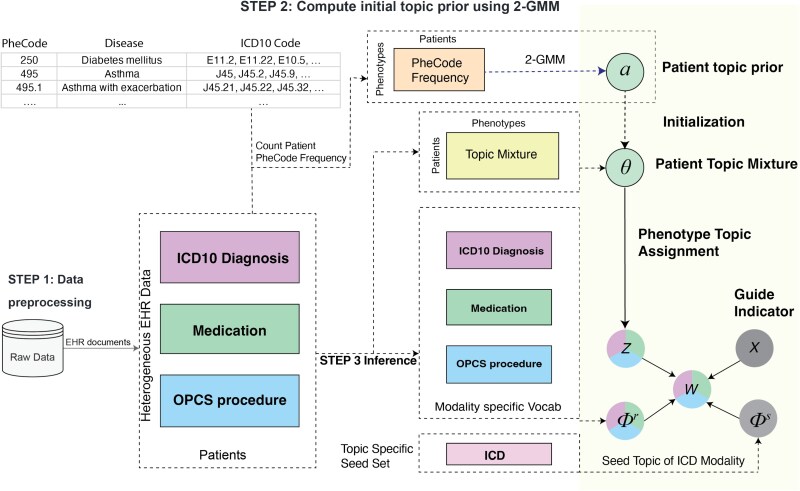
Schematic view of MixEHR-SAGE on the UKB data. In step 1, MixEHR-SAGE ingests patients’ multi-modal EHR data as input. In step 2, MixEHR-SAGE initializes patient topic mixture $\theta $ by fitting 2-component GMM to PheCode count matrix. In step 3, MixEHR-SAGE performs variational inference to infer latent variables including seed topics $\phi _{k}^{s(m=\mathrm{ICD})}$ and regular topics $\phi _{k}^{r(m=\mathrm{ICD})}$ for ICD modality, regular topics for other unguided modalities $\phi _{k}^{r(m)}$, and patient topic mixture $\theta $. $W$ represents EHR observations, and $Z$ represents the latent topic assignment.

MixEHR-SAGE consists of three key steps: (i) assembling a multi-modal EHR dataset (i.e. ICD codes, medications, procedures, etc.) with modality-specific bag-of-words representations; (ii) fitting mixture models to patients’ PheCode counts to estimate topic prior probabilities for each reference phenotype; and (iii) inferring the posterior distributions of latent variables using a collapsed variational inference algorithm (Fig. 1). In Section MixEHR-SAGE discovers interpretable topics from UKB data, we first evaluated the quality of phenotype topics inferred by MixEHR-SAGE in the UKB. In Section MixEHR-SAGE accurately predicts incident diagnoses from baseline characteristics, we assessed MixEHR-SAGE phenotype topics’ ability to predict incident diagnoses based on baseline EHR features in the UKB. In Section MixEHR-SAGE identifies novel genetic associations, we performed genetic association analyses using derived continuous disease risk scores leading to the identification of novel and meaningful genetic associations.

### MixEHR-SAGE details

We consider each patient’s medical records as a document indexed by $d \in \{1, \ldots , D \}$. Each patient’s phenotype topic mixture $\theta _{d}$ is generated from a $K$-dimensional Dirichlet distribution with a prior parameter $\alpha $. For a document of size $N_{d}$, each EHR observation (e.g. an ICD code) is treated as a word $w_{di}^{(m)}$, where $i \in \{1, \ldots , N_{d} \}$ indexes the word and $m \in \{1, \ldots , M \}$ indicates the data modality. Each word $w_{i}^{(m)}$ is associated with a latent topic assignment $z_{di} \in \{1, \dots ,K\}$.

MixEHR-SAGE incorporates expert-defined PheWAS mapping as domain knowledge to guide topic inference and improve clinical interpretability. Specifically, PheWAS projects each PheCode to a set of clinically related ICD codes, allowing us to infuse phenotype concepts directly into MixEHR-SAGE. As a result, MixEHR-SAGE can learn over 1000 phenotype topics, each corresponding to an expert-defined phenotype concept. For the expert-guided ICD modality, MixEHR-SAGE utilizes seed-guided topic mechanism [14] to represent each phenotype topic $k \in \{1, \ldots , K \}$ with two distributions: a seed-topic distribution $\phi _{k}^{s(m=\mathrm{ICD})}$ over only its seed set $V_{k}^{m = \mathrm{ICD}}$ and a regular-topic distribution $\phi _{k}^{r(m=\mathrm{ICD})}$ over the entire feature vocabulary $V^{m = \mathrm{ICD}}$. The seed topics capture the phenotype-specific expert knowledge, while the regular topics govern the general phenotype distributions. Each ICD code $w_{di}^{(m=\mathrm{ICD})}$ is drawn from a mixture distribution: $x_{di} \phi _{z_{di}=k} ^{s(m=\mathrm{ICD})} + (1-x_{di}) \phi _{z_{di}=k} ^{r(m=\mathrm{ICD})}$, where $x_{di}$ indicates whether an ICD code $w_{di}^{(m=\mathrm{ICD})}$ is generated from a seed topic ($x_{di} = 1$) or a regular topic ($x_{di} = 0$). The indicator $x_{di}$ follows a Bernoulli distribution with the seed-topic rate $\pi _{k}$, representing the probability that an ICD code is drawn from the $k$th seed topic.

In contrast, a non-ICD EHR code $w_{di}^{(m \neq \mathrm{ICD})}$ (e.g. medications, procedures) is only sampled from the regular topic $\phi _{z_{di}=k}^{r(m \neq \mathrm{ICD})}$. Although these unguided modalities do not directly benefit from the incorporated expert knowledge, MixEHR-SAGE shares PheCode-guided information through the patient topic mixture $\theta $.

The implementation of the proposed MixEHR-SAGE model comprises three key steps:

1. **Multi-modal EHR data construction**: We processed the multi-modal EHR dataset comprising ICD codes, medications, and Office of Population Censuses and Surveys (OPCS-4) procedures into three matrices (Fig. 1, step 1). A detailed description of the UKB data processing pipeline is provided in Section UK Biobank data processing.
2. **Initialization of phenotype topic priors**: For each phenotype, we fit a two-component Gaussian mixture model (GMM) to patients’ PheCode counts to estimate the topic prior $\alpha _{k}$ (Fig. 1, step 2). Further details on this procedure are provided in Section Initialization of phenotype topic priors.
3. **Posterior approximate inference**: We infer the posterior distributions of latent variables using a mean-field, collapsed variational inference algorithm (Fig. 1, step 3). The full mathematical formulation and derivation of this inference algorithm are provided in Section Collapsed variational inference algorithm.

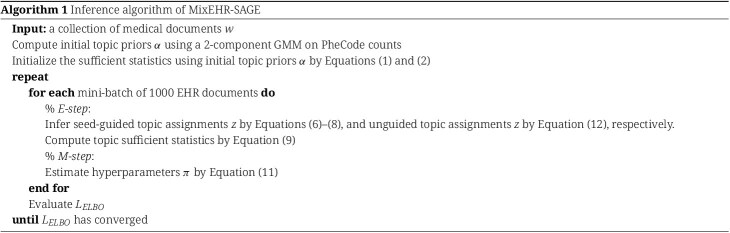



The complete inference algorithm is summarized in Algorithm 1. To effectively handle large-scale EHR data, we performed stochastic variational inference using mini-batches containing 1000 EHR documents per batch [15]. We used the validation set to fine-tune the topic hyperparameters $\mu $ and $\beta $ to minimize the held-out negative log-likelihood. Upon convergence of the ELBO, we can compute the collapsed variables $(\theta , \phi ^{r}, \phi ^{s})$ with their respective variational expectations:


(1) \begin{align*} & \mathbb{E}_{q}[\theta_{dk}] = \frac{\mathbb{E}_{q}[m_{dk}] + \alpha_{dk}} {\mathbb{E}_{q}[m_{d.}] + \sum_{k}^{K} \alpha_{dk}},\quad \mathbb{E}_{q}[\phi_{wk}^{r(m)}] = \frac{\mathbb{E}_{q}[n_{wk}^{(m)}] + \beta} {\mathbb{E}_{q}[n_{.k}^{(m)}] + \beta V^{(m)}} \nonumber \\ & \mathbb{E}_{q}[\phi_{wk}^{s(m=\mathrm{ICD})}] = \frac{\mathbb{E}_{q}[s_{wk}^{(m=\mathrm{ICD})}] + \mu}{\mathbb{E}_{q}[s_{.k}^{(m=\mathrm{ICD})}] + \mu V_{k}^{(m=\mathrm{ICD})}}\end{align*}

Here, $m_{dk}$ denotes the sufficient statistic for the topic mixture of patient $d$, and $m_{d\cdot }$ is its summation over all topics. For sufficient statistic of topic assignments, $n_{wk}$ indicates the number of times word $w$ is assigned to regular topic $k$, and $s_{wk}$ indicates the number of times seed word $w$ is assigned to seed topic $k$. Additionally, $n_{\cdot k}$ and $s_{\cdot k}$ are the total counts of regular words and seed words assigned to topic $k$ across all patients, respectively. The notations of variables are defined in Supplementary Table S2. The full derivation of the inference algorithm is provided in Section Collapsed variational inference algorithm.

### UK Biobank data processing

The UKB is a prospective population cohort comprising $\sim $500 000 participants aged 40–69 at recruitment [16]. At the recruitment visit, participants provided informed consent and answered questions on socio-demographic and health-related factors, including medication use. Hospitalization data were obtained through the Hospital Episode Statistics dataset, with records included up to January 2022. To enable large-scale genomic analysis, genome-wide genetic variants were assessed in all participants using the UKB Axiom Array that measures $\sim $850 000 variants. Genotype imputation procedure using the Haplotype Reference Consortium, the UK10k and the 1000 Genomes Consortium as a haplotype reference panel allowed over 90 million variants to be imputed.

**Figure 2:**
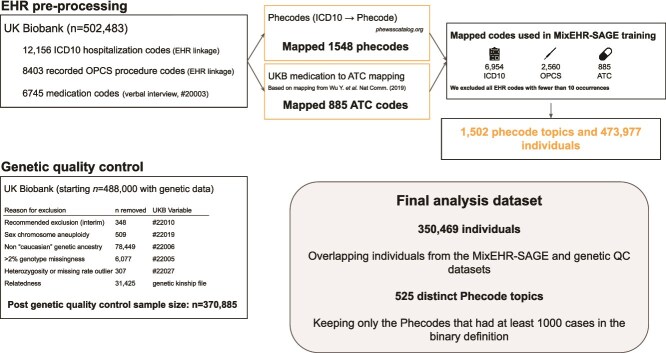
Overview of the UKB data QC steps used to train MixEHR-SAGE, following exclusion criteria during genetic QC, including non-European ancestry, excess missing rate, and relatedness.

An overview of EHR data preprocessing and quality control (QC)is provided in Fig. 2. We utilized three data modalities from the UKB to train MixEHR-SAGE. We included ICD-10 diagnostic codes corresponding to both primary and secondary causes of hospitalization. We also included self-reported medication codes (variable #20003), collected during verbal interviews with a research nurse conducted during any assessment center visit to capture pharmacotherapy data as a second modality. As the third modality, we incorporated OPCS-4 codes capturing surgical and operative procedures. We excluded any EHR code with <10 occurrences. We mapped 6745 unique drug codes in UKB to 885 Anatomical Therapeutic Chemical (ATC) codes using a published mapping method, grouping related drugs together into clinically meaningful therapeutic categories [17]. The processed UKB data included 6954 ICD-10 codes and 2560 OPCS-4 codes.

### Inferring expected phenotypes

The probability of disease incidence was calculated based on the generative process of latent Dirichlet allocation [18]. Specifically, we inferred the expected patient-by-ICD matrix by multiplying the patient topic mixture $\theta \in \mathbb{R}^{D\times K}$ with the topic distribution matrix $\phi \in \mathbb{R}^{K\times V^{ICD}}$. The inferred phenotype probabilities are at low numerical scale because it is derived from Dirichlet distribution with dimensionality equal to the total number of phenotypes (i.e. over 500). To be comparable with the binary phenotype and also improve GWAS analysis, we sought to rescale the inferred phenotype probabilities while maintaining the relative difference among individuals. Specifically, for each inferred ICD code, we fit a simple logistic regression treating the binary ICD code $j$ for patient $d$ as the response variable and the inferred ICD probabilities as the input feature:


(2) \begin{align*} \beta^{*}_{j},b^{*}_{j} \leftarrow \underset{\beta_{j}, b_{j}}{\arg\min} -y_{d,j}\log\hat{y}_{d,j} - (1-y_{d,j})\log(1-\hat{y}_{d,j})\end{align*}

where $\hat{y}_{d,j} = 1/(1+\exp (-\tilde{y}_{d,j}\beta _{j} + b_{j}))$ and $\tilde{y}_{d,j}=\theta _{d,.}\phi _{.,j}$. We then aggregated ICD codes onto their corresponding PheCodes, resulting in a Patient-by-PheCode matrix with 1213 unique PheCodes. The expected sample sizes for each PheCode $k \in \{1, \ldots , K \}$ under the new matrix are calculated by summing its columns across all patients $D$.

### Genetic quality control

Prior to performing GWAS, we conducted genotype QC to ensure the reliability of the genetic data. This involved applying several filtering steps to exclude individuals based on genotyping quality metrics, ancestry, and relatedness (Fig. 2).

We first excluded individuals marked as Recommended Genomic Analysis Exclusions (variable #22010, $n$ = 348) due to poor heterozygosity or high missing rate. Additionally, we excluded those with sex chromosome aneuploidy (variable #22019, $n$ = 509). To account for population stratification, we excluded individuals of non-European genetic ancestry identified by genotype-based PC Analysis (variable #22006, $n$ = 78 449). We further excluded individuals with >2% genotype missingness (variable #22005, $n$ = 6077), as high levels of missing data may indicate poor genotyping quality. We additionally removed individuals identified as outliers in heterozygosity and missingness rate (variable #22027, $n$ = 307), as substantial deviations from the expected distribution may reflect genotyping errors or sample contamination. Finally, because logistic regression assumes independent samples, we excluded related individuals to prevent statistical inflation from genetic relatedness. Specifically, we removed one individual at random from each pair with a kinship coefficient >0.0884 (variable #22021, $n$ = 31 425). After applying all genetic QC criteria, the final dataset included 370 885 unrelated individuals of European ancestry with high-quality genotyping data, ensuring a robust foundation for association analysis.

### Association analysis

To assess genetic associations across phenotypes, we performed GWAS using PLINK 1.9 [19]. We applied two statistical models for GWAS: (i) logistic regression using binary phenotypes based on PheCode definitions, adjusting for age, sex, and 20 PCs as covariates; and (ii) linear regression using continuous disease risk scores inferred from MixEHR-SAGE while adjusting for the same covariates. For both models, we applied standard GWAS QC procedures described in Section Genetic quality control. To define independently associated genetic loci, we used linkage disequilibrium (LD) clumping as implemented in PLINK v1.9. We clumped variants based on a primary significance threshold of $P<5 \times 10^{-8}$, a clumping window of 1000 kb around each index SNP, and an LD cutoff of $r^{2}=0.2$.

### External validation

To perform external validation, we compared our type 2 diabetes (T2D)-associated loci with those reported by Suzuki *et al.* [20], a large-scale T2D meta-analysis including 428 452 cases from the Type 2 Diabetes Global Genetics Initiative (T2DGGI). Lead SNPs for independent loci were defined separately in our UKB analysis and the European subset of the T2DGGI study via the same LD-based clumping method 2.6. Padding regions of $\pm 500$ kb were added to the lead SNPs to compare significantly associated loci from both studies using bedtools 2.31.0 [21]. For visualization of regional association signals, we generated locus-specific plots using LocusCompare, which facilitates side-by-side comparison between the two studies while incorporating LD structure from the 1000 Genomes EUR reference panel [22].

## Results

### MixEHR-SAGE discovers interpretable topics from UKB data

**Figure 3:**
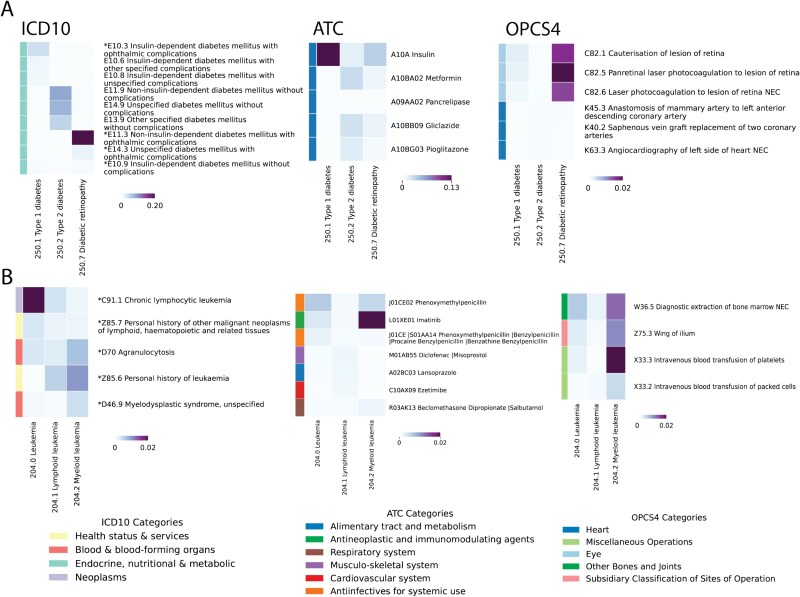
Top 3 highest-probability EHR codes for T2D and leukemia inferred by MixEHR-SAGE. The selected phenotypes belong to diabetes (A) and leukemia (B) categories. For every phenotype, the top 3 EHR codes per modality are presented with duplication removed. For the ICD modality, the asterisk symbols (*) indicate “regular ICD codes” for the corresponding phenotypes. The colorbar represents the probability derived from topic distribution $\phi _{dk}$.

To ensure the broad applicability of our proposed method, we evaluated MixEHR-SAGE’s ability to capture meaningful phenotype topics from the EHR data in UKB dataset (Fig. 1). To validate the interpretability of the inferred disease topics, we extracted the top ICD 10 codes, ATC, and OPCS-4 codes with the highest probabilities for the six selected phenotype topics, focusing on two case studies involving diabetic diseases and leukemia cancers, respectively (Fig. 3).

The top 3 EHR codes exhibit strong clinical relevance to the selected phenotypes. The topic for Type 1 Diabetes (T1D) includes key insulin-dependent conditions namely “E10.3 insulin-dependent diabetes with ophthalmic complications,” “E10.6 T1D mellitus with other specified complications,” and “E10.8 T1D mellitus with unspecified complications” (Fig. 3A). In contrast, the top three ICD-10 codes identified for the T2D topic are “E11.9 Non-insulin-dependent diabetes mellitus,” “E14.9 Unspecified diabetes mellitus,” and “E11.3 Non-insulin-dependent diabetes with ophthalmic complications,” which correspond to different T2D subtypes. For diabetic retinopathy (250.7), “E11.3 Type 2 diabetes mellitus with ophthalmic complications” is the top diagnosis code, which then leads to common microvascular complications, along with E14.3 and E10.9. In the medication modality, the prominent ATC codes are insulin (A10A) for T1D, metformin (A10BA02), the first-line treatment for T2D, as well as gliclazide (A10BB09) and pioglitazone (A10BG03), both of which are commonly used to control blood sugar levels [23, 24].

While diabetes itself is typically managed with medications like insulin or metformin as its first-line therapy, its complications, such as diabetic retinopathy, often require surgical treatments. Indeed, diabetic retinopathy is more strongly linked to ophthalmologic procedures such as laser photocoagulation for retinal lesions (C82.5 andC82.6) and cauterization of lesion of retina (C82.1) than T1D and T2D. These procedures are standard treatments for proliferative diabetic retinopathy, helping to prevent vision loss [25]. T2D was also identified with other complications, such as anastomosis of the artery (K45.3), and saphenous vein graft replacement of two coronary arteries (K40.2). T2D is strongly associated with increased risk of cardiovascular events like coronary artery disease (CAD) [26], which makes angiocardiography crucial for diagnosing and assessing the severity of CAD in diabetic patients [27].

For the leukemia topic 204.0, “C91.1 chronic lymphocytic leukemia,” “Z85.7 history of lymphoid neoplasms,” and “D70 agranulocytosis” are the three top ICD 10 codes (Fig. 3B). The first two are essentially the diagnostic rule of the cancer. “D70 agranulocytosis” is a condition of severely reduced white blood cells that has been shown to increase infection risk and could potentially develop into leukemia [28, 29]. For the myeloid leukemia topic (204.2), “D46.9 myelodysplastic syndrome” is one of the top ICD codes that aligns with growing recognition of myelodysplastic syndrome (MDS) as a preleukemic state that progresses to acute myeloid leukemia in $\sim 30\%$ of cases [30, 31]. For the ATC medication modality, MixEHR-SAGE highlighted imatinib (L01XE01), the hallmark targeted therapy for chronic myeloid leukemia, alongside diclofenac (M01AB55), a nonsteroidal anti-inflammatory drug shown to induce apoptosis in acute myeloid leukemia (AML) cell lines [32, 33]. Besides direct treatment, MixEHR-SAGE also identified phenoxymethylpenicillin (J01CE02), underscoring the routine use of antibiotics to treat infections common in leukemia patients. For the OPCS treatment procedure modality, MixEHR-SAGE identified bone marrow extraction (W36.5) that is important for disease diagnosis. Additionally, intravenous platelet transfusion (X33.2) and packed red blood cell transfusion (X33.2) are used to raise platelet counts and control bleeding [34, 35]. Together, these findings demonstrate MixEHR-SAGE’s ability to recover a comprehensive, clinically coherent picture of leukemia care.

To assess whether phenotype topics are dominated by generic or nonspecific ICD codes, we quantified topic purity, defined as the proportion of a topic’s top-5 ICD codes that map to the same ICD-10 organ-system chapter. Across all topics, the mean ICD purity is 0.62, indicating that the highest-weight codes typically arise from one major physiological system. Overall, $64\%$ of topics achieve purity $\geq 0.60$ and $15\%$ reach purity $\geq 0.90$, while fewer than $4.5\%$ show mixed-system codes. Notably, ICD codes outside a topic’s dominant chapter are often clinically meaningful comorbidities, complications, or diagnostic extensions. For example, the dementia topic (PheCode: 290.1) is primarily composed of codes “Delirium superimposed on dementia” (F05.1), “Unspecified dementia” (F03), “Dementia in Alzheimer disease, unspecified” (F00.9), “Dementia in other specified diseases including Parkinson’s disease” (F02.8) and also include “G30.9” for Alzheimer’s disease. The summaries for ICD topics are provided in Supplementary Table S3.

Similar patterns were observed across the medication and procedure modalities. Medication topics have an average purity of 0.54, with $48\%$ achieving purity $\geq 0.60$, and procedure topics have an average purity of 0.58, with $58\%$ reaching $\geq 0.60$. In both cases, the highest-weight codes align with expected pharmacologic classes or procedural categories. Complete purity summaries for all topics with purity $\geq 0.60$ are provided in Supplementary Tables S4 and S5.

We then examined relationships between phenotype topics using both phenotype similarity and phenotype comorbidity analyses. Phenotype similarity was defined as the Pearson correlation between topic–feature distributions ($\phi _{dk}$), reflecting topics that share similar clinical code patterns. Phenotype comorbidity was defined as the Pearson correlation between patient-level topic mixtures ($\theta $), capturing topics that tend to co-occur within individuals. For six representative diseases, including T2D, diabetic retinopathy, hypercholesterolemia, CAD, asthma, and essential hypertension, we visualized the top-5 most similar phenotype topics and the top-5 most comorbid topics. Further information are provided in Supplementary Fig. S3.

To further validate MixEHR-SAGE’s ability to produce interpretable disease topics, we applied it to the MIMIC-III dataset [36]. Besides ICD and medication modalities, MixEHR-SAGE also inferred interpretable phenotype topics across Current Procedural Terminology, Diagnosis Related Group, lab tests, and doctor notes. These results demonstrate its ability to leverage diverse EHR modalities for comprehensive phenotype characterization. Further details for MIMIC-III applications are provided in Section Qualitative phenotyping evaluation in the MIMIC-III dataset, and further information for other disease categories of UKB data are provided in Supplementary Fig. S4.

### MixEHR-SAGE accurately predicts incident diagnoses from baseline characteristics

**Figure 4:**
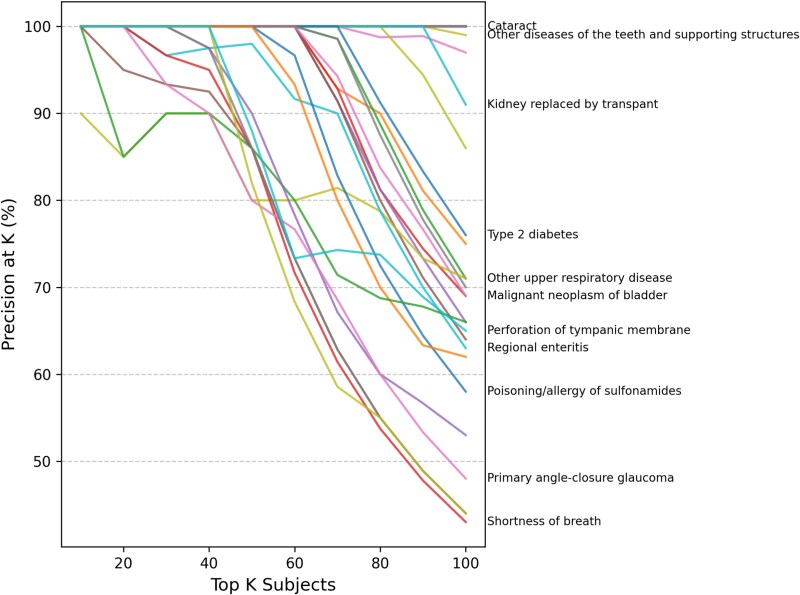
Disease risk prioritization of UKB individuals using patient-level topic mixture inferred by MixEHR-SAGE, showing precision as a fraction of number of incident cases among top $K$ patients, where , with sustained high precision $K \in \{10, \ldots , 100\}$ across $K$-values for diseases such as T2D and cataracts.

**Figure 5:**
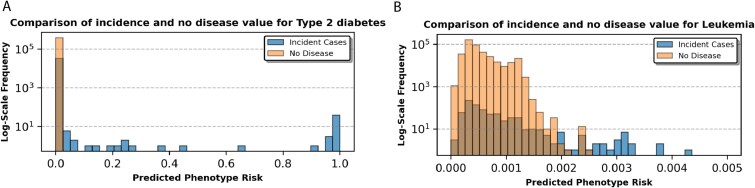
Predicted phenotype risk and clinical features for T2D and leukemia case studies, comparing bar plots of the prevalence (y-axis) of the predicted phenotype risk scores (x-axis) between incident cases and the control groupfor (A) T2D and (B) leukemia.

To evaluate the predictive performance of the inferred topics for clinically meaningful events, we assessed their ability to predict incident diagnoses using baseline characteristics from the UKB. We analyzed 320 253 individuals using data from their first visit. Prevalent events were the events occurring prior to the recruitment visit, based on hospital inpatient data. In contrast, incident events referred to new inpatient diagnoses recorded after the baseline visit, with no prior record of the matching PheCode before the individual’s baseline date [37]. For controls of each target disease, we applied PheCode exclusion criteria to remove individuals with the related PheCode [8]. We evaluated the top-$K$ highest-risk individuals identified by MixEHR-SAGE, with $K$ ranging from 10 to 100. For each target disease, we ranked patients based on their predicted risk scores derived from patient topic mixture (i.e. $\theta _{dk}$ in (1)) (Fig. 4). We observe that individuals with an incident diagnoses had higher average predicted risk scores than the controls. In addition to the main incidence prediction analysis, we conducted a subsampling experiment to assess how sample size influences the predictive performance of MixEHR-SAGE. We trained the model on random subsets of the UKB cohort ($10\%, 50\%, 80\%$, and $100\%$) and evaluated phenotype prediction accuracy using the same precision metric. Performance increased consistently with sample size. Detailed results are provided in Supplementary Fig. S5. Using T2D and leukemia as case studies, we identified the top 10 patients ($K$ = 10) with the highest predicted risk scores for each disease. We examined their most frequently associated phenotypes, medications, and surgical procedures to assess the key features contributing to model prediction. The predicted risk distributions for incident cases and controls demonstrated a right-skewed pattern among individuals, who later developed T2D and leukemia. Therefore, MixEHR-SAGE can effectively identify high-risk individuals prior to their future clinical diagnosis (Fig. 5A and B).

**Figure 6:**
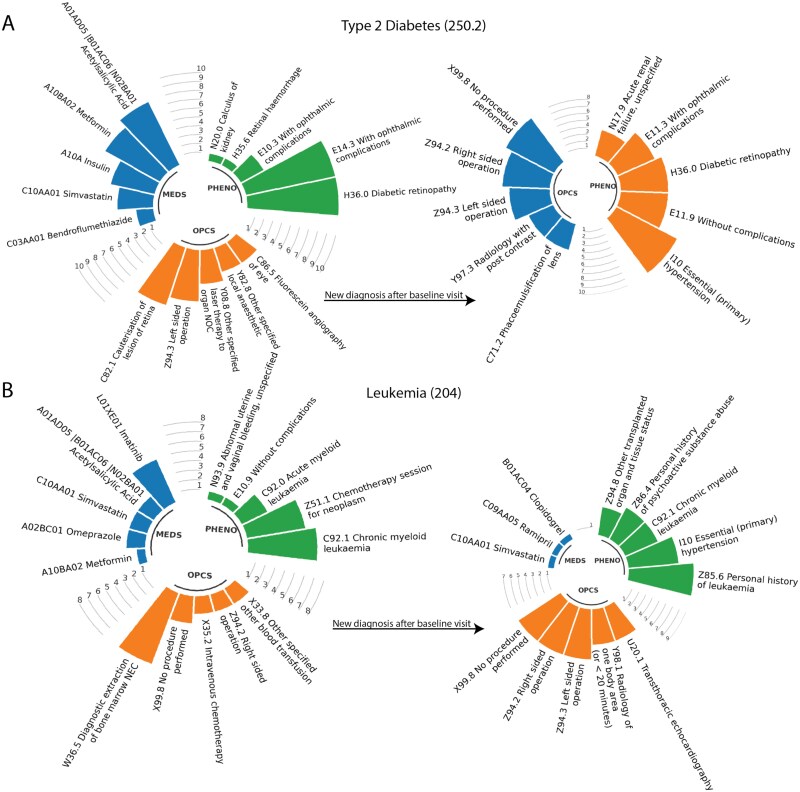
Top k patients and their most frequent EHR codes before and after baseline visits. The circular barplot illustrates the top 5 most frequent EHR codes from the Medications (MDES), PheCodes (PHENO), and OPCS modalities observed among the top 10 high-risk patients inferred by MixEHR-SAGE before and after their baseline visits. (A) T2D and (B) Leukemia.

To evaluate the clinical relevance of the estimated T2D risk scores, we analyzed high-risk individuals identified by MixEHR-SAGE before and after the baseline assessment. Concordant to the above topic analysis (Section MixEHR-SAGE discovers interpretable topics from UKB data), among those high-risk individuals, some patients had already been diagnosed with T2D-related complications (e.g. diabetic retinopathy), prescribed antidiabetic medications (e.g. insulin and metformin), and received relevant procedures associated with diabetic retinopathy like C82.1 and C86.5 (Fig. 6A). Although these individuals were not T2D cases based on PheCodes before the baseline visit, our model assigned them high-risk scores for the T2D topic, demonstrating its utility for early diagnosis. After the baseline visit, individuals were diagnosed with T2D, either with or without complications (E11.3 and E11.9). Notably, a significant number of patients were also diagnosed with essential hypertension (I10), a common condition in the UKB population and a well-known comorbidity of T2D (Fig. 6A, right). Newly diagnosed procedures, such as phacoemulsification of the lens (C71.2), were associated with diabetic retinopathy and also with patients diagnosed with T2D and ophthalmic complications. A recent study reviewed the safety of this surgery for T2D patients, and it is considered a safe treatment with careful management [38].

For Lymphoid Leukemia (PheCodes 204), many high-risk individuals identified by MixEHR-SAGE already exhibited pre-existing conditions related to leukemia before the baseline visit. Risk estimation was primarily driven by the occurrence of leukemia-related phenotypes and procedures (Fig. 6B, left panel). In particular, procedures such as diagnostic extractions of bone marrow, also refers to as bone marrow biopsies (W36.5) and intravenous chemotherapy (X35.2) suggest that these individuals had prior hematologic abnormalities or were evaluated for related conditions through surgical procedures [34]. Some of the high-risk patients of Lymphoid Leukemia were diagnosed with Chronic Myeloid Leukemia (ICD code C92.1) either at the baseline visit or at the follow-up visit, which maps to PheCode 204.22 rather than 204 (Fig. 6B, right panel). These results demonstrate MixEHR-SAGE’s ability to capture both broad hematological disease categories and specific leukemia subtypes. Additionally, some individuals in the high-risk group were later diagnosed with a different leukemia subtype, highlighting MixEHR-SAGE’s ability to capture shared risk factors across various hematologic malignancies.

In summary, T2D prediction was strongly driven by medication use and prebaseline diagnostic codes, resulting in a distinct separation between incident cases and nondisease individuals (Fig. 6B). In contrast, leukemia prediction was primarily influenced by pre-existing hematological conditions, resulting in more overlap in risk distributions between incident cases and nondisease individuals (Fig. 6A). Overall, MixEHR-SAGE accurately identified high-risk individuals and enabled risk stratification by capturing prevalence and incidence of clinical events from the multi-modal EHR data.

### MixEHR-SAGE identifies novel genetic associations

**Figure 7:**
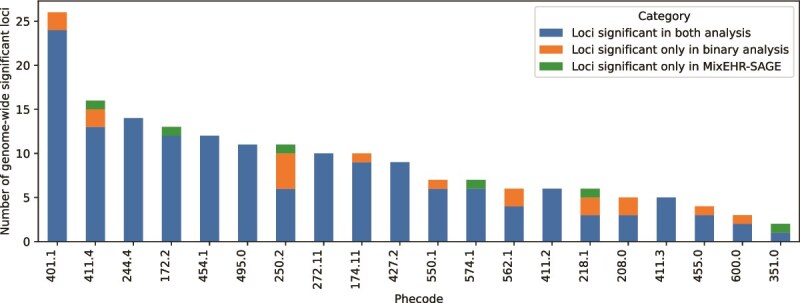
Summary of the number of loci reaching genome-wide significance ($P<5 \times 10^{-8}$) in the binary Phecode and MixEHR-SAGE models. The 20 most common diseases according to the Phecode definitions (i.e. most prevalent) are shown, sorted by the total number of identified loci. The loci are defined using LD-based clumping. The six phenotypes (and their Phecodes) with the largest number of genome-wide significant loci detected using MixEHR-SAGE are: coronary atherosclerosis (411.4; $n$ loci = 16), other nonepithelial cancer of skin (172.2; $n$ loci = 13), T2D (250.2, $n = 7$), cholelithiasis (574.1; $n$ loci = 7), uterine leiomyoma (218.1; $n$ loci = 4), and other peripheral nerve disorders (351; $n$ loci = 2).

Building on the incidence prediction results, we conducted GWAS across 525 phenotypes, each of which has >1000 cases, using both the traditional binary disease labels and our continuous disease risk scores inferred from MixEHR-SAGE. The majority of genome-wide significant loci were identified by both approaches, and MixEHR-SAGE further uncovers novel loci in several phenotypes (Fig. 7). Note that our GWAS analyses are restricted to predominantly European-ancestry participants in the UKB. The identified associations may not generalize to other ancestry groups. Replication in more diverse cohorts will be important to assess the portability of MixEHR-SAGE–derived risk scores and GWAS findings in the future.

To showcase MixEHR-SAGE’s utility across phenotypes with different prevalence counts, we chose two contrasting examples: T2D and Leukemia. T2D ranks among the top 20 most common diagnoses in UKB. In contrast, leukemia (204) has only 178 cases in UKB but emerged as one of the top 10 phenotypes that show the greatest gain in its expected sample size ($n = 221$). The comparison of genome-wide significant loci identified by MixEHR-SAGE and the traditional binary GWAS for the 100 most prevalent diseases is provided in Supplementary Table S6.

**Figure 8:**
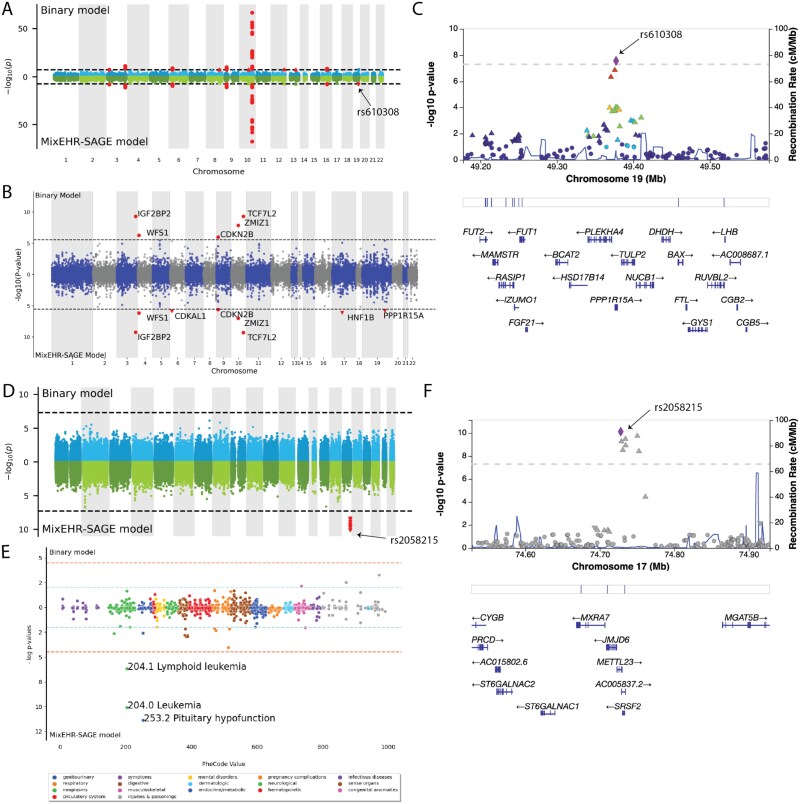
Genetic signals linked to T2D (A–C) and leukemia (D–F) using MixEHR-SAGE topics and PheCode labels. (A) Genome-wide comparison of genetic associations for T2D based on binary PheCodes (top) and MixEHR-SAGE topics (bottom), revealing new findings on chromosome 19. (B) Gene-level analysis of T2D GWAS showing the significantly associated genes ($P \leq .05/17549 = 2.849^{-6}$). (C) A zoomed-in plot of chromosome 19 of a T2D locus showing the location of the significant loci and its nearby genes. (D) Genome-wide comparison of genetic associations for leukemia using binary PheCode labels (top) and MixEHR-SAGE-inferred topics (bottom). MixEHR-SAGE successfully identified new loci on the chromosome 17. (E) The phenome-wide association analysis for the leukemia locus harboring genetic variant rs2058215. This reveals significant association of 2 types of leukemia, namely lymphoid leukemia and leukemia. (F) Locus Zoom plot of the rs2058215-locus, showing the significant hits and its closest genes.

For T2D, we identified a locus harboring *PPP1R15A*, which was missed by the binary approach (Fig. 8A–C). The lead variant of the locus is rs610308, a previously identified T2D variant [3]. The rs610308 variant is a missense variant of *PPP1R15A* (Protein Phosphatase 1 Regulatory Subunit 15A) based on dbSNP [39]. This gene plays a critical role in cellular stress responses and insulin signaling pathways, and is essential for metabolic regulation and pancreatic beta-cell function [40, 41].

This demonstrates that MixEHR-SAGE can identify genetic variants associated with both T2D and its complications (e.g. diabetic kidney disease). In addition, we also identified two more well-established T2D loci that were missed by the binary model, including *HNF1B* and *CDKAL1* [42, 43] (Fig. 8B).

To replicate our findings independently, the T2D-associated loci identified by our approach are compared with those reported in the multi-ancestry meta-analyses of Suzuki *et al*. [20]. All identified loci were successfully replicated in this large study when using windows defined from LD clumped lead SNPs using the method described in Section External validation. In some instances, the lead variants appeared to differ between the two approaches (Supplementary Fig. S7). This is likely due to differences in LD patterns between the ancestral backgrounds present in our cohort and the multi-ancestry meta-analysis panel, or due to differences in the statistical power to detect specific variants.

Importantly, the fact that our MixEHR-SAGE infers continuous phenotype probabilities for each individual enables performing GWAS on some of the low-prevalent phenotypes such as leukemia, for which the number of cases is fewer than 1000. As a result, the traditional binary phecode-based GWAS analysis is unable to detect any GWAS locus due to the lack of power. On the other hand, our approach identified a significant locus, which was missed by the conventional binary method (Fig. 8D). This association signal was led by the leading SNP rs2058215. When performing PheWAS on this leading SNP, MixEHR-SAGE also indicated its association with lymphoid leukemia (204.1) (Fig. 8E). The locus harbors *JMJD6*, *METTL23*, and *MFSD11*, as well as a 3’ UTR variant of *SRSF2* (Fig. 8F). The gene *JMJD6* is implicated in various cancers, including leukemia, due to its versatile role in epigenetic regulation, RNA splicing, and histone modification [44]. It contributes to tumor development mainly through regulating immune responses and cancer-related pathways. It also plays a crucial role in the differentiation of hematopoietic stem cells, a critical process in leukemia pathogenesis [44]. *SRSF2* (Serine and arginine-rich splicing factor 2) is known to regulate gene expression essential for normal hematopoiesis. Mutations in *SRSF2* have been strongly associated with various myeloid malignancies, including AML, MDS, and chronic myelomonocytic leukemia [45, 46]. Functional studies show that SRSF2 mutations alter hematopoietic stem cell differentiation, leading to clonal expansion of preleukemic cells. Furthermore, *SRSF2* mutations frequently co-occur with mutations in epigenetic regulators such as *TET2*, *ASXL1*, *IDH2*, and *DNMT3A*, suggesting a synergistic effect in leukemic transformation [47–51]. Given its key role in RNA processing and leukemia pathogenesis, *SRSF2* is being increasingly explored as a therapeutic target, with current efforts focusing on small molecules that modulate RNA splicing [52]. In addition, rs2058215 is in high LD ($r^{2} = 0.69$) with rs9897202, an eQTL SNP for *MXRA7* based on whole blood samples from GTEx [53]. *MXRA7* is involved in hematopoiesis and immune response and serves as a possible effector gene on leukemia risk [54]. A recent study demonstrated that *MXRA7* could influence the pathogenesis of acute promyelocytic leukemia through regulation of cell differentiation [55].

The FinnGen study is a large-scale genomics initiative that has analyzed over 500 000 Finnish biobank samples and correlated genetic variation with health data to understand disease mechanisms and predispositions. The project is a collaboration between research organizations and biobanks within Finland and international industry partners [56]. To further validate the leukemia-associated JMJD6/SRSF2 locus discovered by MixEHR-SAGE, we examined external association data from FinnGen, where JMJD6 region variants show significant association with leukemia-related phenotypes, including lymphoid leukemia ($P = 8.1\times 10^{-4}$, $OR = 3.30$, $n = 1945$), and chronic lymphocytic leukemia ($P = 3.1\times 10^{-3}$, $OR = 15.02$, $n = 303$). Additionally, a MAGMA gene-based test conducted on the MixEHR-SAGE leukemia summary statistics identified strong gene-level signals for SRSF2 ($P=2.43\times 10^{-7}$) and MFSD11 ($P=9.06\times 10^{-10}$), supporting the robustness of this locus [57].

Together, by modeling disease risk on a continuum via MixEHR-SAGE, we capture genetic signals often missed by the conventional GWAS that relies on the binary disease labels.

## Discussion

In this study, we introduce MixEHR-SAGE, a seed-guided topic model designed to improve the phenotypic representation and discovery of novel GWAS loci. By integrating PheCode-guided prior knowledge, MixEHR-SAGE enables the inference of >1000 interpretable phenotype topics, each aligned with clinically defined disease concepts. A key property of MixEHR-SAGE is its ability to integrate multiple data modalities (e.g. ICD codes, medications, and procedures), thereby capturing more comprehensive and clinically meaningful disease representations. Unlike traditional phenotyping methods that solely rely on binary case-control definitions, MixEHR-SAGE models diseases as continuous risk scores, reflecting the spectrum of phenotype severity. This approach is particularly beneficial for diseases with varying severity levels, subtypes, or progression stages, such as metabolic disorders and cancer.

We applied MixEHR-SAGE to the UKB dataset, demonstrating its ability to infer interpretable phenotype topics and improve GWAS discovery. The inferred topics are highly interpretable even for modalities without explicit expert knowledge, such as medications and procedures. We then utilized each patient’s topic mixture as continuous disease risk scores to predict incident disease onset. MixEHR-SAGE accurately identified high-risk individuals for developing T2D and leukemia. For T2D, MixEHR-SAGE captured early pharmacotherapy signals such as metformin prescriptions; for leukemia, relevant ICD diagnostic and OPCS procedure codes were leveraged. Through GWAS analysis, we identified novel genetic signals complementary to those found by traditional binary models. For T2D, MixEHR-SAGE revealed genetic associations specifically linked to clinically relevant complications such as diabetic retinopathy and diabetic kidney diseases. For leukemia, MixEHR-SAGE identified a novel loci harboring *JMJD6* that was missed by the binary approach.

There are several future directions to be explored.

In this study, MixEHR-SAGE is applied only to categorical EHR codes (i.e. diagnoses, procedures, and medications), and does not incorporate continuous measurements such as lab results or vital signs. This is particularly crucial for phenotypes like T2D or leukemia where lab results (e.g. HbA1c and white blood cell counts) carry rich information. However, extending MixEHR-SAGE to handle continuous data is nontrivial, because classify topic models assume multinomial-Dirichlet likelihoods for discrete data, whereas lab results are continuous. One possible extension is to bin normalized continuous measurements into discrete categories; another is to extend MixEHR-SAGE with a separate Gaussian likelihood for continuous features, and jointly model discrete codes and continuous measurements within a unified multi-view topic model. We expect that such joint modeling of discrete EHR codes and continuous lab results would further improve phenotype accuracy for lab-intensive diseases.

Another potential limitation relates to the uncertainty of the continuous risk scores inferred by MixEHR-SAGE. While the model produces posterior distributions over patient-topic proportions we used point estimates for downstream risk scores and GWAS analyses in the current study. Recent work demonstrates that incorporating uncertainty-aware phenotypes into downstream analyses can improve robustness and interpretability, and similar approaches could be integrated into MixEHR-SAGE as future work [58].

Although PheCodes provide a widely used and clinically motivated mapping of diagnosis codes, they are not without limitations. Certain conditions are less reliably captured by the existing PheCode definitions, thereby reducing the interpretability of seed-guided topics for those phenotypes. Importantly, MixEHR-SAGE is designed to remain robust in such settings: when seed information is noisy or incomplete, the model naturally relies more heavily on its unguided, data-driven topics learned across all EHR modalities. As a result, even for phenotypes poorly represented in PheCodes, MixEHR-SAGE continues to discover coherent structure in the data (Fig. 3). Nevertheless, because our model jointly infers ATC medication and treatment procedure topics alongside diagnosis-driven topics, inaccuracies in PheCode definitions can indirectly affect the downstream interpretability of these modality-specific topics. While our current study focuses on phenotypes that are well represented by existing PheCodes, future extensions that incorporate improved phenotype definitions or high-resolution clinical labels may further strengthen topic interpretability across all modalities.

Unlike purely unsupervised models, which can identify *de novo* phenotype clusters without predefined labels, MixEHR-SAGE uses PheCode-seeded priors to guide topic inference. This improves interpretability and ensures alignment with known disease categories. Although MixEHR-SAGE topics can expand or deviate from their initial seeds, novel clusters are less likely to emerge compared with unsupervised models. Future research could explore a hybrid approach, allocating a portion of topics for unguided discovery while seeding others for interpretability, similar to MixEHR-Nest, which is a hierarchical guided-topic modeling approach to infer subphenotypes from multi-modal EHR data [59].

A key challenge in genetic studies is its restricted generalizability across diverse populations. Although the UKB dataset includes a diverse cohort, it predominantly consists of individuals of European ancestry, limiting the generalizability of our findings to multi-ethnic populations. Since genetic architecture and disease prevalence vary by ancestry, future work could extend MixEHR-SAGE to more diverse cohorts. Additionally, we excluded rare diseases due to limited sample sizes, and future work could explore adapting MixEHR-SAGE for low-prevalence conditions. Although MixEHR-SAGE demonstrated improved genetic associations, it did not explore polygenic risk score (PRS) prediction. Future work could explore novel PRS models using probabilistic phenotype representations that reflect continuous disease severity rather than binary case definitions.

Another direction for future research is incorporating longitudinal modeling and survival analysis into MixEHR-SAGE. While MixEHR-SAGE effectively stratifies disease phenotypes, it does not explicitly model disease progression and predict survival outcomes. Many chronic diseases, including cancer, cardiovascular conditions, and neurodegenerative disorders, exhibit dynamic progression patterns that could be better captured by time-to-event models. Incorporating survival analysis methods (e.g. Cox proportional hazards models or deep learning-based survival models) into MixEHR-SAGE framework could enhance phenotype risk stratification for chronic diseases. Recent advancements like MixEHR-SurG, which demonstrated promising results on Quebec CHD and MIMIC-III datasets, could be used to extend MixEHR-SAGE framework to analyze survival outcomes in the UKB dataset [60]. Additionally, by integrating the inferred phenotype topics with Mendelian randomization could help identify causal relationships between genetic variants and disease progression mechanisms. Lastly, federated learning or privacy-preserving EHR modeling is another emerging challenge/opportunity [61].

## Conclusion

Our results demonstrate the value of probabilistic topic modeling for multi-modal EHR phenotyping to improve GWAS discovery. MixEHR-SAGE offers a scalable framework capable of phenotyping over 1000 phenotypes and identifying novel genetic associations beyond traditional binary case definitions. As biobanks expand, more diverse data types such as biosignals, exome sequencing, and whole-genome sequencing will become available. Automatic phenotyping approaches such as MixEHR-SAGE are critical for integrating clinical phenotyping with genetic discovery, thereby advancing biomedical informatics and genetic epidemiology.

**Key Points**

- We introduce MixEHR-SAGE, a seed-and-guided multi-modal topic modeling framework for electronic health record (EHR).
- MixEHR-SAGE improves disease incidence prediction compared with conventional models in the UK Biobank and MIMIC-III datasets.
- Our method enables continuous phenotype risk scores, capturing nuanced signals beyond binary disease labels.
- MixEHR-SAGE improves genetic discovery, identifying loci missed by standard genome-wide association studies approaches.
- MixEHR-SAGE is applicable for phenotyping, risk stratification, and genomic association studies in population-scale cohorts.

## Supplementary Material

supplementary-material_bbag030

## Data Availability

This research has been conducted using the UK Biobank Resource (http://www.ukbiobank.ac.uk/) under Application Number 45551. Derived data supporting the findings of this study are available from the corresponding author upon reasonable request.

## Code availability

The custom code and scripts used to implement MixEHR-SAGE, including sample data preprocessing and topic modeling pipelines, are publicly available at GitHub via the following link: https://github.com/li-lab-mcgill/MixEHR-SAGE.
