# Supplementary material for: PheCode-guided multi-modal topic modeling of electronic health records improves disease incidence prediction and GWAS discovery from UK Biobank
Source: Brief Bioinform. 2026 Feb 2;27(1):bbag030. doi: 10.1093/bib/bbag030 (PMC12862981; doi:10.1093/bib/bbag030)
Supplement: supplementary-material_bbag030 [file supplementary-material_bbag030.zip › MixEHR_SAGE_UKB_Supplementary_bbag030.pdf]

1 Supplementary Methods

1.1 Methodological background

MixEHR-Seed model MixEHR-Seed performs seed-guided topic modeling by computing each phenotype topic k of ICD modality with two distributions: a seed-topic distribution $\phi_k^{s(m=\text{ICD})}$ over only its seed set $V_k^{m=\text{ICD}}$ and a regular-topic distribution $\phi_k^{r(m=\text{ICD})}$ over the entire feature vocabulary $V^{m=\text{ICD}}$. The regular topics govern the global phenotype distributions and the seed topics capture the phenotype-specific information.

Each ICD code $w_{di}^{(m=\text{ICD})}$ is drawn from a mixture distribution: $x_{di}\phi_{z_{di}=k}^{s(m=\text{ICD})} + (1-x_{di})\phi_{z_{di}=k}^{r(m=\text{ICD})}$, where x_{di} indicates whether an ICD code $w_{di}^{(m=\text{ICD})}$ is generated from a seed topic ($x_{di}=1$) or a regular topic ($x_{di}=0$). The seed-topic rate π_k controls the sampling probability that an ICD code is drawn from the seed topic rather than the regular topic. In contrast, for other unguided modalities, a non-ICD EHR feature $w_{di}^{(m\neq\text{ICD})}$ is only sampled from the regular topic $\phi_{z_{di}=k}^{r(m\neq\text{ICD})}$. Although other unguided modalities do not directly benefit from expert knowledge, MixEHR-Seed shares guided information through the general patient topic mixture variable θ_d.

In summary, the MixEHR-Seed EHR data generative process is described as follows:

1. For each phenotype topic $k=\{1,\ldots,K\}$ of ICD modality $m=$ ICD:
   (a) Draw regular topic $\phi_k^{r(m=\text{ICD})} \sim \text{Dir}(\beta)$ over the entire ICD vocabulary $V^{m=\text{ICD}}$
   (b) Draw seed topic $\phi_k^{s(m=\text{ICD})} \sim \text{Dir}(\mu)$ over only the topic-specific seed set $V_k^{m=\text{ICD}}$
   (c) Draw seed-topic rate $\pi_k \sim \text{Beta}(1,1)$
2. For each phenotype topic $k=\{1,\ldots,K\}$ of other unguided modalities $m\neq$ ICD:
   (a) Draw regular topic $\phi_k^{r(m\neq\text{ICD})} \sim \text{Dir}(\beta)$ over the entire feature vocabulary $V^{(m\neq\text{ICD})}$
3. For each EHR document $d=\{1,\ldots,D\}$:
   (a) Draw phenotype topic proportion from topic prior $\theta_d \sim \text{Dir}(\alpha)$
   (b) For each EHR observation $i=\{1,\ldots,N_d\}$, if $w_{di}^{(m=\text{ICD})}$ is an ICD code:
      i. Draw topic assignment $z_{di} \sim \text{Mult}(\theta_d)$
      ii. Draw seed-topic indicator $x_{di} \sim \text{Bern}(\pi_{z_{di}})$.
      iii. Draw an ICD code:

$$w_{di}^{(m=\text{ICD})} \sim \begin{cases} \text{Mult}(\phi_{z_{di}}^{s(m=\text{ICD})}), \text{if } x_{di}=1 \\ \text{Mult}(\phi_{z_{di}}^{r(m=\text{ICD})}), \text{otherwise} \end{cases}$$

   (c) For each EHR observation $i=1,\ldots,N_d$ under other unguided modalities $m\neq$ ICD:
      i. Draw topic assignment $z_{di} \sim \text{Mult}(\theta_d)$
      ii. Draw an EHR observation:

$$w_{di}^{(m\neq\text{ICD})} \sim \text{Mult}(\phi_{z_{di}}^{r(m\neq\text{ICD})})$$

where Dir, Multi, Bern abbreviate Dirichlet, Multinomial, and Bernoulli distributions, respectively.

MixEHR-Guided model MixEHR-G assumes patient topic mixture θ_d is generated from Dirichlet distribution with K-dimensional asymmetric hyperparameters $\alpha_d = (\alpha_{d1}, \ldots, \alpha_{dK})$, which is initialized by a modified Multimodal Automated Phenotyping (MAP) approach [1]. For each phenotype topic k, it estimates prior probabilities by fitting Poisson and Lognormal mixture models to D-dimensional patients' PheCode counts. Consequently, a higher prior likelihood of phenotype presence should generate a proportionally higher probability of feature attribution to that phenotype.

In contrast to MixEHR-Seed, MixEHR-G generates phenotype topics solely based on the regular topic distributions and draws EHR feature $w_{di}^{(m)}$ from these regular topics $\phi_{z_{di}=k}^{r(m)}$.

1. For each phenotype topic $k = \{1, \ldots, K\}$ of any modality $m \in \{1, \ldots, M\}$:
   (a) Draw a phenotype topic $\phi_k^{r(m)} \sim Dir(\beta_{vm})$ over the entire vocabulary $V^{(m)}$
2. For each EHR document $d = \{1, \ldots, D\}$:
   (a) Draw phenotype topic proportion from topic prior $\theta_d \sim \text{Dir}(\alpha_d)$
   (b) For each EHR observation $i = \{1, \ldots, N_d\}$ of any modality $m \in \{1, \ldots, M\}$:
      i. Draw topic assignment $z_{di} \sim \text{Mult}(\theta_d)$
      ii. Draw an EHR observation:

$$w_{di}^{(m)} \sim \text{Mult}(\phi_{z_{di}}^{r(m)})$$

1.2 MixEHR-SAGE methodology

1.2.1 Initialization of phenotype topic priors

For each reference phenotype k, we run a two-component GMM on PheCode frequencies among patients. The normalized posterior probabilities of the higher components from the two components are then utilized as the initial topic prior $\alpha_{dk} = \frac{\alpha_{dk}}{\sum_k^K \alpha_{dk}}$ [2]. In most cases, Patients who have no recorded PheCode occurrence are unlikely to have the disease, thus the topic prior α_{dk} is set to zero for these patients. Based on the calculated topic priors, the sufficient statistics with regards to the ICD modality are initialized as follows:

$$\begin{aligned}
n_{vk}^{(m=\text{ICD})} &= \begin{cases} \sum_d^D \sum_i^{N_d} [w_{di} == v]\alpha_{dk}, & \text{if } w_{di} \text{ is a regular code under topic } k \\ \sum_d^D \sum_i^{N_d} [w_{di} == v]\alpha_{dk}(1-\pi_k), & \text{if } w_{di} \text{ is a seed code under topic } k \end{cases} \\
s_{vk}^{(m=\text{ICD})} &= \sum_d^D \sum_i^{N_d} [w_{di} == v]\alpha_{dk}\pi_k, \quad \text{if } w_{di} \text{ is a seed code under topic } k \\
m_{dk} &= N_d \alpha_{dk}
\end{aligned} \tag{1}$$

where n_{vk} denotes the number of times word v is assigned to a regular topic k, s_{vk} is the number of times the seed word v is assigned to seed topic k, and m_{dk} represents the estimated topic probabilities for each EHR document d.

For the unguided modalities, MixEHR-SAGE only needs to update the sufficient statistics $n_{vk}^{(m \neq \text{ICD})}$:

$$n_{vk}^{(m \neq \text{ICD})} = \sum_{d}^{D} \sum_{i}^{N_d} [w_{di} == v] \alpha_{dk} \tag{2}$$

1.2.2 Collapsed variational inference algorithm

In this section, we present the variational inference algorithm that approximates the posterior distribution of latent variables in the proposed MixEHR-SAGE. To reduce probabilistic dependencies among latent variables, we integrated out the latent variables θ by exploiting the conditional independency in the PGM (Figure 1) and the conjugate relationship between Dirichlet variables θ and multinomial topic assignment variables z. Similarly, we integrated out Dirichlet variables ϕ^r and ϕ^s due to its conjugacy to the multinomial EHR feature variables w. Note that we can always compute the sufficient statistics of these Dirichlet variables given the expectations of the multinomial variables and the hyperparameters (see Eq. (1)). For the K-dimensional hyperparameters π, we used empirical Bayes to optimize their fixed point estimates.

To infer the latent variables z, we applied variational inference by turning our inference problem into an optimization problem over an evidence lower bound (ELBO) of marginal likelihood:

$$L_{ELBO} = \mathbb{E}_q[\log p(w, z, \mid \beta, \mu, \pi)] - \mathbb{E}_q[\log q(z)] \tag{3}$$

Under the independent mean-field assumption, we proposed a fully factorized variational family for the latent variables z and η:

$$q(z) = \prod_{d}^{D} \prod_{i}^{N_d} q(z_{di} \mid \gamma_{di}) \tag{4}$$

where the variational density of z is multinomial. Therefore, we employed mean-field, collapsed variational inference to optimize the variational parameters of $q(z)$.

Regardless of the modality, the variational parameter of the topic assignment γ_{dik} for EHR feature i in EHR document d under phenotype topic k is defined as:

$$\gamma_{dik} \propto \frac{\exp(\mathbb{E}_{q(z^{-di})}[\log p(w, z)])}{\sum_{k=1}^{K} \exp(\mathbb{E}_{q(z^{-di})}[\log p(w, z)])} \tag{5}$$

where the notation $-di$ means the exclusion of token i in record d.

For the ICD modality, we take into account four scenarios when inferring the topic assignment of token i in document d under phenotype topic k:

1. If w_{di} is a seed ICD code under topic k, the posterior probability it is sampled from the *seed* topic distribution of topic k is:

$$\gamma_{dik}^{ss(m=\text{ICD})} \propto (\mathbb{E}_q[m_{dk}^{-di}] + \alpha_{dk}) \cdot \frac{\mathbb{E}_q[s_{wk}^{(m=\text{ICD})-di}] + \mu}{\mathbb{E}_q[s_{.k}^{(m=\text{ICD})-di}] + \mu V_k^{(m=\text{ICD})}} \cdot \pi_k \tag{6}$$

2. If w_{di} is a seed ICD code under topic k, the posterior probability it is sampled from the *regular* topic

distribution of topic k is:

$$\gamma_{dik}^{sr(m=\text{ICD})} \propto (\mathbb{E}_q[m_{dk}^{-di}] + \alpha_{dk}) \cdot \frac{\mathbb{E}_q[n_{wk}^{(m=\text{ICD})-di}] + \beta}{\mathbb{E}_q[n_{.k}^{(m=\text{ICD})-di}] + \beta V^{(m=\text{ICD})}} \cdot (1 - \pi_k) \tag{7}$$

3. If w_{di} is a regular ICD code w.r.t. topic k, the posterior probability it is sampled from the *seed* topic distribution of topic k is 0.

4. If w_{di} is a regular ICD code w.r.t. topic k, the posterior probability it is sampled from the *regular* topic distribution of topic k is:

$$\gamma_{dik}^{rr(m=\text{ICD})} \propto (\mathbb{E}_q[m_{dk}^{-di}] + \alpha_{dk}) \cdot \frac{\mathbb{E}_q[n_{wk}^{(m=\text{ICD})-di}] + \beta}{\mathbb{E}_q[n_{.k}^{(m=\text{ICD})-di}] + \beta V^{(m=\text{ICD})}} \tag{8}$$

where $n_{.k}$ and $s_{.k}$ are simply the summation of the sufficient statistics n_{wk} and s_{wk} over all features. The expected values of sufficient statistics are computed as follows:

$$\mathbb{E}_q[n_{wk}^{(m=\text{ICD})-di}] = \sum_{d' \neq d}^{D} \sum_{i}^{N_{d'}} [w_{d'i} = w](\gamma_{d'ik}^{rr(m=\text{ICD})} + \gamma_{d'ik}^{sr(m=\text{ICD})})$$

$$\mathbb{E}_q[n_{.k}^{(m=\text{ICD})-di}] = \sum_{d' \neq d}^{D} \sum_{i}^{N_{d'}} \gamma_{d'ik}^{rr(m=\text{ICD})} + \gamma_{d'ik}^{sr(m=\text{ICD})}$$

$$\mathbb{E}_q[s_{wk}^{(m=\text{ICD})-di}] = \sum_{d' \neq d}^{D} \sum_{i}^{N_{d'}} [w_{d'i} = w]\gamma_{d'ik}^{ss(m=\text{ICD})}$$

$$\mathbb{E}_q[s_{.k}^{(m=\text{ICD})-di}] = \sum_{d' \neq d}^{D} \sum_{i}^{N_{d'}} \gamma_{d'ik}^{ss(m=\text{ICD})}$$

$$\mathbb{E}_q[m_{dk}^{-di}] = \sum_{i' \neq i}^{N_d} \pi_k \gamma_{di'k}^{ss} + (1 - \pi_k)(\gamma_{di'k}^{sr(m=\text{ICD})} + \gamma_{di'k}^{rr(m=\text{ICD})}) \tag{9}$$

Note that the inference of regular topics by Eq. (8) benefits from guided information through $\mathbb{E}_q[m_{dk}]$, whose calculation relies on both seed topics and regular topics. This interdependency is the driving force behind our guided mechanism. Finally, the variational probabilities are normalized such that $\sum_k^K \gamma_{dik}^{ss(m=\text{ICD})} + \gamma_{dik}^{sr(m=\text{ICD})} = 1$ and $\sum_k^K \gamma_{dik}^{rr(m=\text{ICD})} = 1$:

$$\gamma_{dik}^{ss(m=\text{ICD})} = \frac{\gamma_{dik}^{ss(m=\text{ICD})}}{\sum_k^K \gamma_{dik}^{ss(m=\text{ICD})} + \gamma_{dik}^{sr(m=\text{ICD})}}, \quad \gamma_{dik}^{sr(m=\text{ICD})} = \frac{\gamma_{dik}^{sr(m=\text{ICD})}}{\sum_k^K \gamma_{dik}^{ss(m=\text{ICD})} + \gamma_{dik}^{sr(m=\text{ICD})}}$$

$$\gamma_{dik}^{rr(m=\text{ICD})} = \frac{\gamma_{dik}^{rr(m=\text{ICD}})}{\sum_k^K \gamma_{dik}^{rr(m=\text{ICD})}} \tag{10}$$

The seed-topic rates π are estimated by maximizing the marginal likelihood function under the variational

expectations:

$$\pi = \frac{\sum_d^D \sum_i^{N_d} \gamma_{di}^{ss(m=\text{ICD})}}{\sum_d^D \sum_i^{N_d} \gamma_{di}^{ss(m=\text{ICD})} + \gamma_{di}^{sr(m=\text{ICD})}} \tag{11}$$

To infer latent topic assignments of other unguided modalities, the variational parameter $\gamma_{dik}^{rr(m\neq\text{ICD}))}$ is updated without incorporating expert-guided knowledge:

$$\gamma_{dik}^{rr(m\neq\text{ICD})} \propto (\mathbb{E}_q[m_{dk}^{-di}] + \alpha_{dk}) \cdot \frac{\mathbb{E}_q[n_{wk}^{(m\neq\text{ICD})-di}] + \beta}{\mathbb{E}_q[n_{.k}^{(m\neq\text{ICD})-di}] + \beta V^{(m\neq\text{ICD})}} \tag{12}$$

This variational update relies on *document-level* sufficient statistics $\mathbb{E}_q[m_{dk}]$ so that the topic inference for the unguided modalities also benefits from seed-guided information.

$$\mathbb{E}_q[n_{wk}^{(m\neq\text{ICD})}] = \sum_d^D \sum_i^{N_d} [w_{di} = w] \gamma_{dik}^{rr(m\neq\text{ICD})}, \quad \mathbb{E}_q[n_{.k}^{(m\neq\text{ICD})}] = \sum_w^V \mathbb{E}_q[n_{wk}^{(m\neq\text{ICD})}] \tag{13}$$

The complete inference algorithm is summarized in Algorithm 1. To effectively handle large-scale data, we performed stochastic variational inference using mini-batches containing 1,000 EHR documents per batch [3]. We used the validation set to fine-tune the topic hyperparameters μ and β to minimize the held-out negative log-likelihood. Upon convergence of the ELBO, we can compute the collapsed variables (θ, ϕ^r, ϕ^s) with the respective variational expectations:

$$\mathbb{E}_q[\theta_{dk}] = \frac{\mathbb{E}_q[m_{dk}] + \alpha_{dk}}{\mathbb{E}_q[m_{d.}] + \sum_k^K \alpha_{dk}}, \quad \mathbb{E}_q[\phi_{wk}^{r(m)}] = \frac{\mathbb{E}_q[n_{wk}^{(m)}] + \beta}{\mathbb{E}_q[n_{.k}^{(m)}] + \beta V^{(m)}}$$
$$\mathbb{E}_q[\phi_{wk}^{s(m=\text{ICD})}] = \frac{\mathbb{E}_q[s_{wk}^{(m=\text{ICD})}] + \mu}{\mathbb{E}_q[s_{.k}^{(m=\text{ICD})}] + \mu V_k^{(m=\text{ICD})}} \tag{14}$$

We set model hyperparameters following the prior works [4]. By design, the number of topics K is fixed to the number of PheCodes as each PheCode defines a unique phenotype topic. The Dirichlet prior α is initialized using the PheCode-informed strategy described in Section 1.2.1. For the remaining hyperparameters for data generative process, we set a Dirichlet prior $\beta = 0.1$ for regular topics, $\mu = 0.05$ for seed ICD topics, and a seed topic weight $\pi = 0.7$.

1.3 MIMIC-III analysis

1.3.1 MIMIC dataset

MIMIC-III is a clinical database that contains data from 38,597 adult patients and 7870 neonates admitted to the intensive care unit (ICU) of the Beth Israel Deaconess Medical Center between 2001 and 2012. The MIMIC-III dataset comprises a heterogeneous collection of EHR data such as diagnoses, laboratory test results, procedures, medications, and clinical notes for a total of 53,423 distinct hospital admissions [5]. The dataset can be downloaded from the PhysioNet portal and used in accordance with the PhysioNet user agreement. This step allows for a rigorous assessment of MixEHR-SAGE's ability to infer meaningful phenotype structures before its application to large-scale biobank data.

1.3.2 Qualitative phenotyping evaluation in the MIMIC-III dataset

We qualitatively evaluated the inferred topic on the MIMIC-III dataset. We selected 9 common diseases for analysis: asthma (495), congestive heart failure (CHF, 428), chronic obstructive pulmonary disease (COPD, 496), diabetes (250), epilepsy (345), HIV (71), hypertension (401), ischemic heart disease (IHD, 411) and schizophrenia (295.1). For each phenotype, we extracted top 3 EHR features with highest probabilities across modalities (Figure S1A, C). We also compared the inferred disease topic from the MixEHR-Guided model to evaluate topic quality (Figure S1B, D).

We found that the inferred phenotype topics ϕ^r confer high probabilities to seed ICD codes for the selected phenotypes, indicating that expert-guided topic inference helps discover clinically meaningfully disease topics. For instance, the top ICD-9 codes for the schizophrenia topic were its seed ICD codes, 295.90, 295.70, and 295.30, matched the seed codes defined by its corresponding PheCode. The CHF topic assigned high probabilities to ICD codes 428.23, 428.22, 428.21, corresponding to complications of systolic heart failure. Another key finding was that MixEHR-SAGE assigned highest probabilities to non-seed ICD codes, revealing additional clinically relevant features. For example, the asthma topic assigned high probability to esophageal reflux, consistently with epidemiological and Mendelian randomization studies linking childhood-onset asthma to increased risk of gastroesophageal reflux disease [6,7]. Similarly, the hypertension topic assigned high probability to the non-seed ICD code chronic kidney disease not otherwise specified, suggesting it commonly co-occurs with hypertension in ICU patients. We also found that high-probability ICD codes were generally consistent within the same disease systems. For example, 8 of the 9 top codes for CHF, hypertension and IHD, fell under the circulatory system category (Figure S1A).

Furthermore, MixEHR-SAGE inferred clinically meaningful phenotype topics for medication codes, even without using seed codes for this modality (Figure S1C). For example, the top drug codes for diabetes topics included insulin, commonly used to manage blood glucose in diabetic patients. We also identified a top medication prednisone, a corticosteroid used to decrease inflammation in asthma and COPD. Additional high-probability codes from other unguided modalities—-such as Current Procedural Terminology (CPT), Diagnosis Related Group (DRG), lab test, and doctor notes-—are shown in Figure S2.

We also compared the inferred topics from the baseline MixEHR-G method (Figure S1B, D). Most of the top ICD-9 codes were seed codes, while some frequently occurring non-seed ICD codes were incorrectly assigned high probabilities across unrelated disease topics. For instance, the non-seed ICD code 401.9 (Hypertension NOS) appeared with high probability in unrelated diseases like schizophrenia or epilepsy. This issue was more obvious for the medication modality, where unrelated drug codes like saline solutions were assigned high probabilities across nearly all disease topics, indicating the less specificity in the inferred topics from MixEHR-Guided (Figure S1D).

References

[1] Liao, K., Sun, J., Cai, T. *et al.* High-throughput multimodal automated phenotyping (map) with application to phewas. *Journal of the American Medical Informatics Association* **26**, 1255–1262 (2019).

[2] Ahuja, Y. *et al.* sureLDA: A multidisease automated phenotyping method for the electronic health record. *Journal of the American Medical Informatics Association* **27**, 1235–1243 (2020). URL `https://doi.org/10.1093/jamia/ocaa079`. `https://academic.oup.com/jamia/article-pdf/27/8/1235/34153254/ocaa079.pdf`.

[3] Hoffman, M. D., Blei, D. M., Wang, C. & Paisley, J. Stochastic variational inference. *Journal of Machine Learning Research* **14**, 1303–1347 (2013). URL `http://jmlr.org/papers/v14/hoffman13a.html`.

[4] Song, Z., Hu, Y., Verma, A., Buckeridge, D. L. & Li, Y. Automatic phenotyping by a seed-guided topic model. In *Proceedings of the 28th ACM SIGKDD Conference on Knowledge Discovery and Data Mining*, KDD '22, 4713–4723 (Association for Computing Machinery, New York, NY, USA, 2022). URL `https://doi.org/10.1145/3534678.3542675`.

[5] Johnson, A. *et al.* Mimic-iii, a freely accessible critical care database. *PubMed* (2016). URL `https://pubmed.ncbi.nlm.nih.gov/27219127`.

[6] Freuer, D., Linseisen, J. & Meisinger, C. Asthma and the risk of gastrointestinal disorders: A Mendelian randomization study. *BMC Medicine* **20**, 82 (2022).

[7] Mays, E. E. Intrinsic Asthma in Adults: Association With Gastroesophageal Reflux. *JAMA* **236**, 2626–2628 (1976).

Supplementary information

S1 Supplementary Tables

Modality	Admissions	# of Patient	# of Word Token	# of Records
**ICD9-CM code**	First Admit	46509	6555	463195
	Full Admit	46520	6985	651047
**Prescription**	First Admit	38534	9401	1651047
	Full Admit	39363	10450	2010638
**ICD9-CPT code**	First Admit	41591	1926	183429
	Full Admit	42214	1995	215793
**DRG code**	First Admit	46493	3269	77356
	Full Admit	46511	3495	97729
**Lab Test**	First Admit	32104	570	213830
	Full Admit	46252	729	27872575
**Doctor Note**	First Admit	45994	69444	12710608
	Full Admit	46139	69445	16250075

Table S1: Summary of first and full admit data for processed MIMIC-III database

Notations	Descriptions
D	total number of EHR documents in dataset
N_d	total number of features for EHR document d
M	total number of modality in dataset
K	number of phenotype topics
V^m	feature vocabulary for modality m in dataset
$V_k^{(m=\text{ICD})}$	set of seed ICD codes for phenotype topic k
w_{di}	feature index of token i in EHR document d
z_{di}	topic assignment for feature $w_{di^{(m)}}$
x_{di}	binary seed-topic indicator of ICD code $w_{di}^{(m=\text{ICD})}$
$\alpha \in R^{D\times K}$	Dirichlet topic priors
$\theta \in R^{D\times K}$	topic mixture memberships
$\phi^r \in R^{K\times V}$	regular topic distributions
$\phi^s \in R^{K\times S}$	seed topic distributions
$\pi \in R^K$	seed-topic rates
η	topic hyperparameter for regular topics ϕ^r
μ	topic hyperparameter for seed topics ϕ^s

Table S2: Notations in MixEHR-SAGE

‘

Table S3: Topic purity (ICD-10 modality) for all phenotype topics with purity ≥ 0.6. The full table is provided as a CSV file named "MixEHR-SAGE_ICD_Topic_Purity.csv" in the supplementary materials.

Table S4: Topic purity (ATC medication modality) for all phenotype topics with purity ≥ 0.6. The full table is provided as a CSV file named "MixEHR-SAGE_Med_Topic_Purity.csv" in the supplementary materials.

Table S5: Topic purity (OPCS-4 procedure modality) for all phenotype topics with purity ≥ 0.6. The full table is provided as a CSV file named "MixEHR-SAGE_OPCS_Topic_Purity.csv" in the supplementary materials.

Table S6: Genome-wide significant loci identified by MixEHR-SAGE versus traditional binary GWAS approach for the top 100 most prevalent phenotypes. The table was provided as a CSV file named "MixEHR-SAGE_vs_Binary_Loci_Comparison" in the supplementary materials.

Table S7: Topic coherence and topic diversity (top-3 codes) for MixEHR-SAGE vs. LDA baselines across ICD, ATC, and OPCS modalities.

Metrics	Method	ICD	ATC	OPCS
Topic Coherence	MixEHR-SAGE	0.188	0.126	0.166
	LDA (50 topics)	0.114	0.075	0.094
	LDA (100 topics)	0.106	0.072	0.083
Topic Diversity (N=3)	MixEHR-SAGE	0.137	0.052	0.076
	LDA (50 topics)	0.091	0.041	0.052
	LDA (100 topics)	0.070	0.038	0.043

Table S8: Mean LLM-rated topic coherence scores (1–5) for MixEHR-SAGE and LDA baselines over ICD, ATC, and OPCS topics using the top 5 codes.

LLM coherence score (top 5 codes)	ICD	ATC	OPCS
MixEHR-SAGE	3.96	2.48	4.07
LDA (50 topics)	3.10	1.48	2.54
LDA (100 topics)	2.86	1.40	2.26

S2 Supplementary Figures

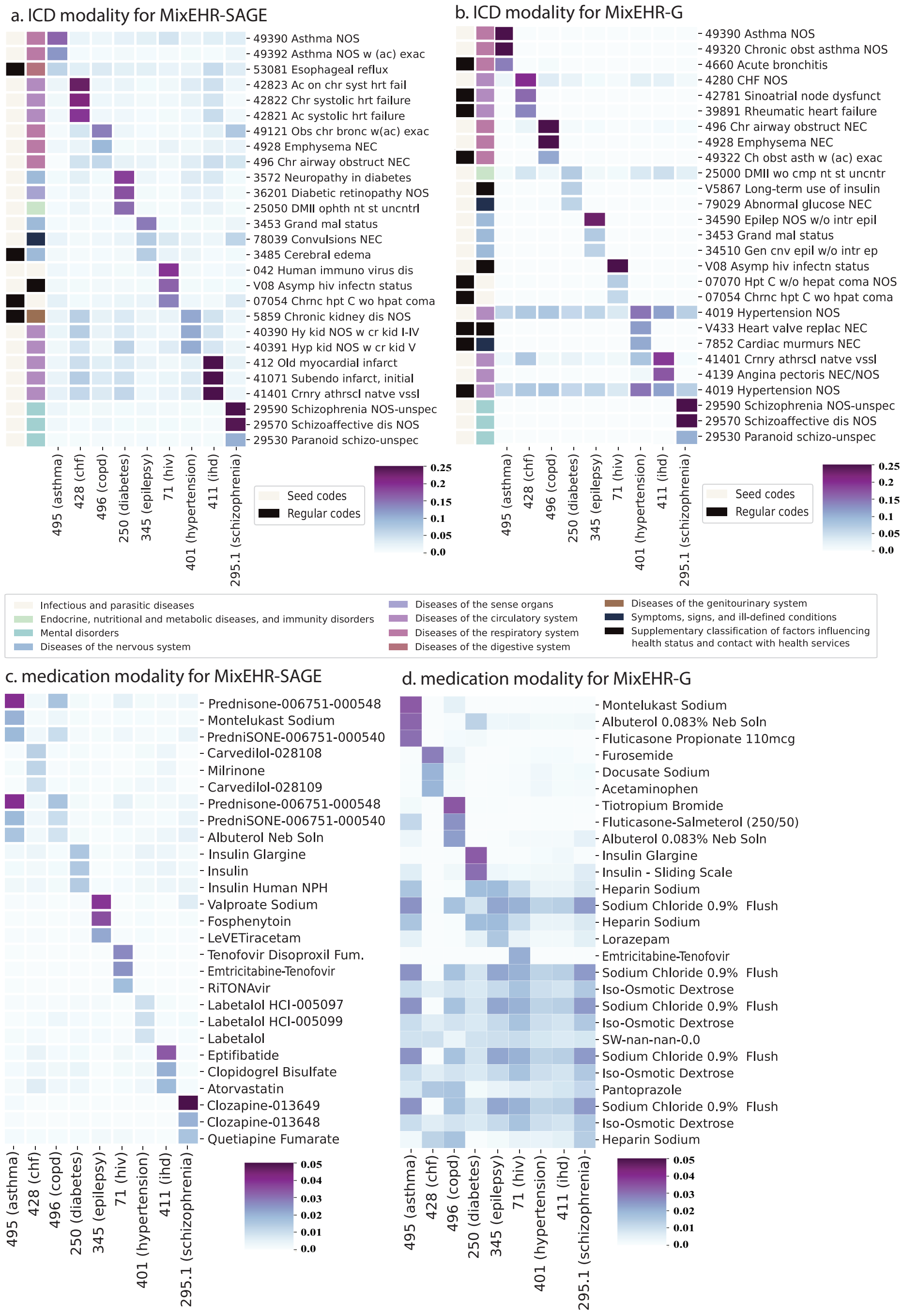

Figure S1: **The 3 highest probability ICD and drug codes for topics inferred by MixEHR-SAGE and MixEHR-Guided on MIMIC-III data.** The top ICD diagnostic codes are presented for (a) MixEHR-SAGE and (b) MixEHR-Guided. The colorbars represent different ICD categories and whether an ICD code is a seed or regular code. The top 3 drugs for the 9 selected phenotype topics as identified by (c) MixEHR-SAGE and (d) MixEHR-Guided

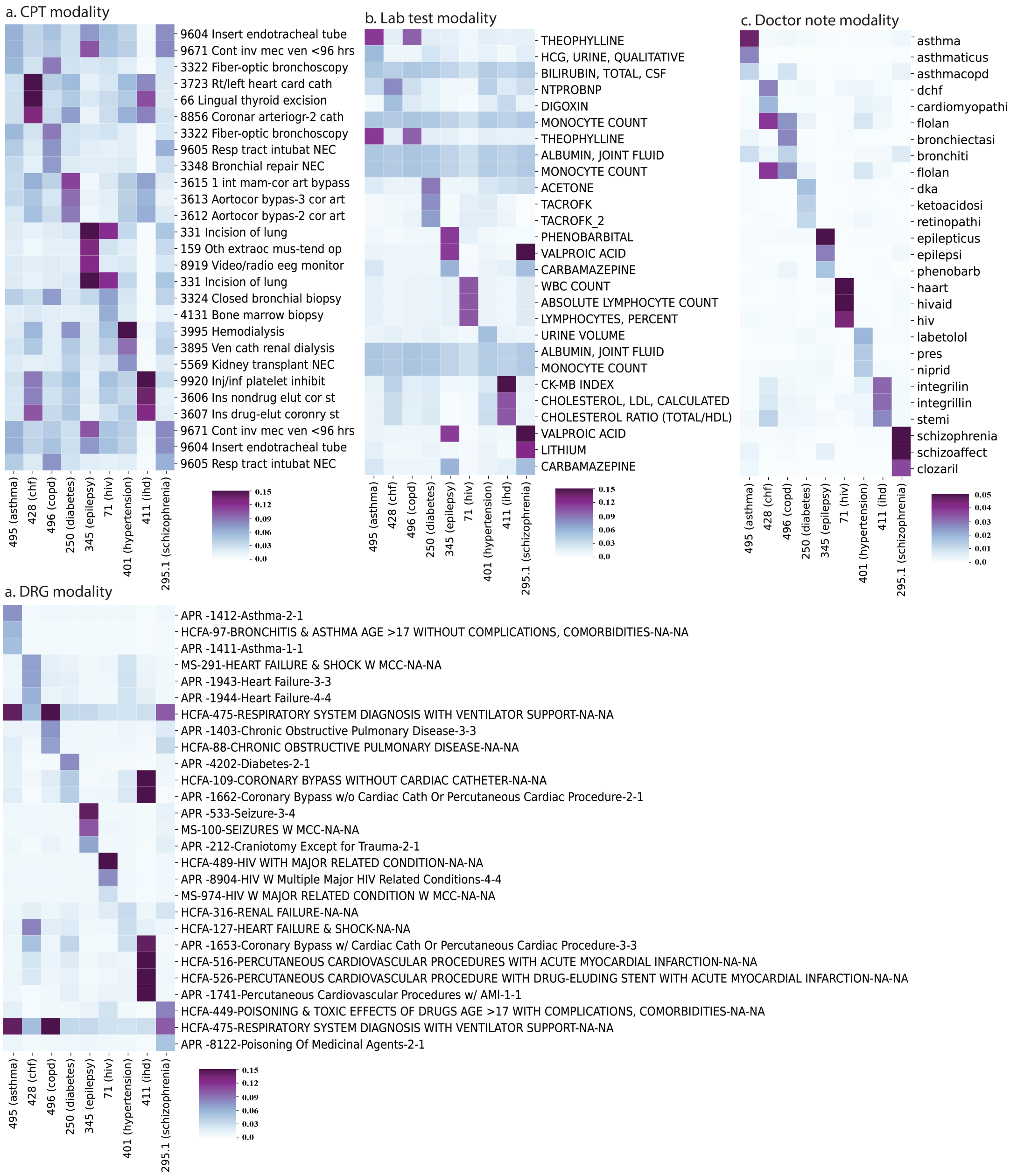

Figure S2: **The 3 highest probability EHR codes (CPT, DRG, lab tests and doctor notes) for topics inferred by MixEHR-SAGE on MIMIC-III data**

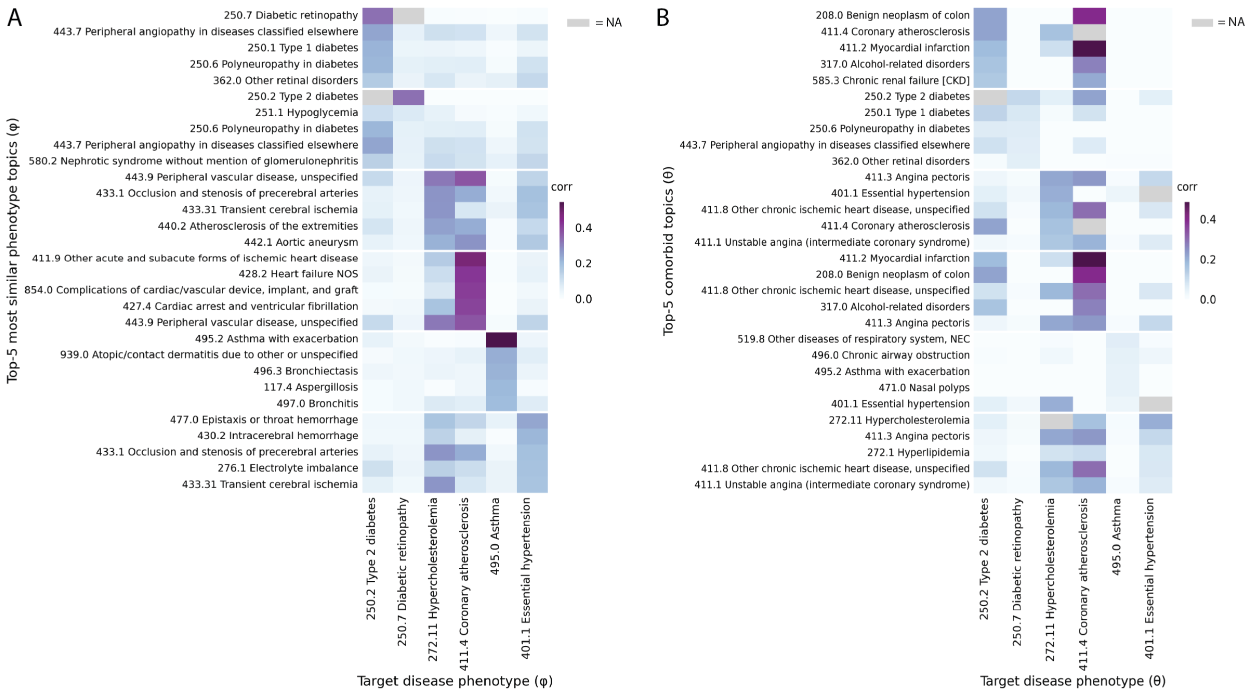

Figure S3: **Predicted similarities and comorbidities of 6 diverse phenotypes. (a) 5 most similar parent phenotype topics for each of 6 diverse disease phenotypes, as predicted by Spearman correlations among MixEHR-SAGE's topic distributions over all multimodal EHR features ϕ. (b) 5 most comorbid phenotype topics per target disease as predicted by Spearman correlations among MixEHR-SAGE's patient-topic mixtures θ. Self-correlations were masked as grey.**

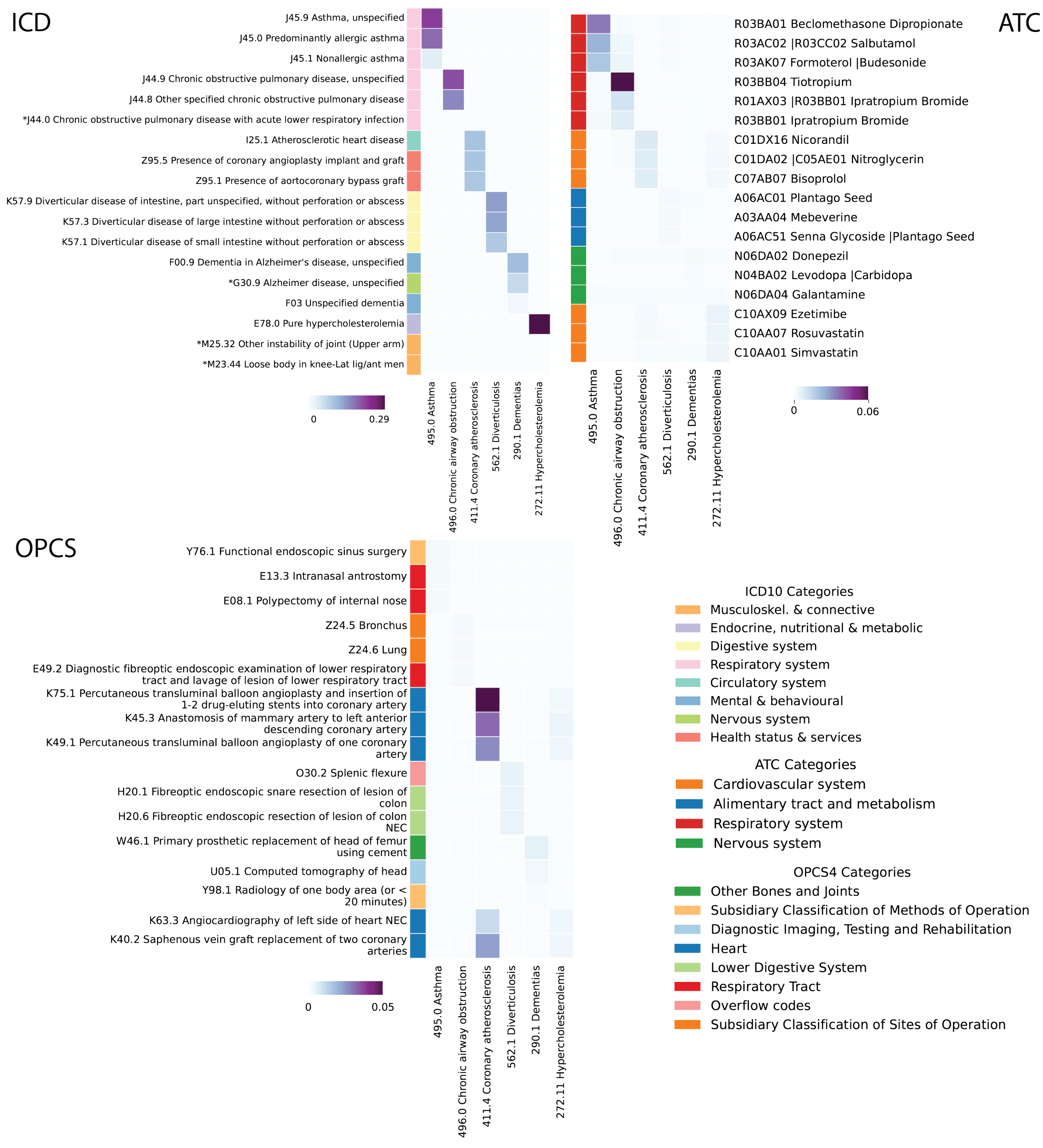

Figure S4: **The 3 highest probability EHR codes (ICD, ATC, OPCS4 code) for different topics identified by MixEHR-SAGE on UKB data.**

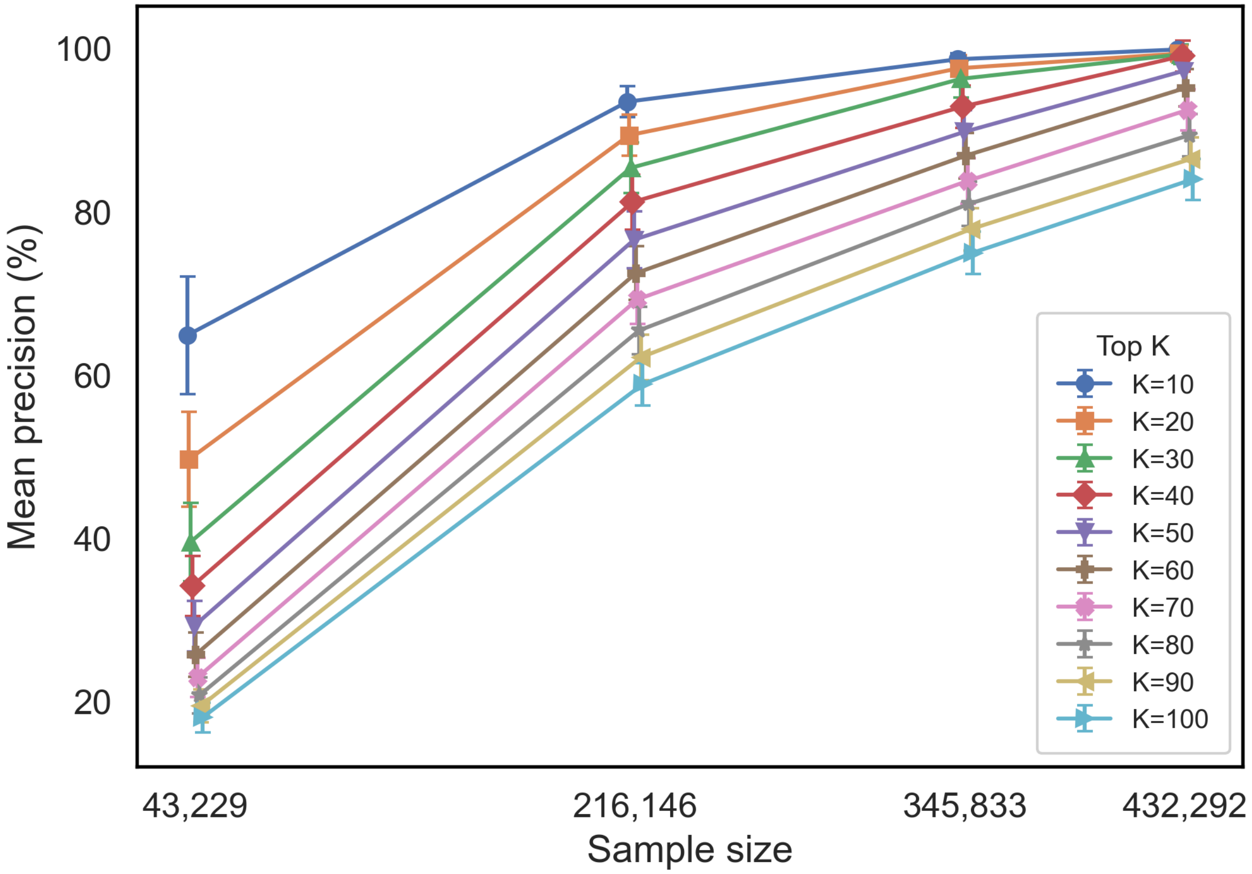

Figure S5: **Incidence prediction performance across different subsampling rates. Average precision at K, where** $K \in \{10, \ldots, 100\}$ **is computed over the top 50 PheCode topics.**

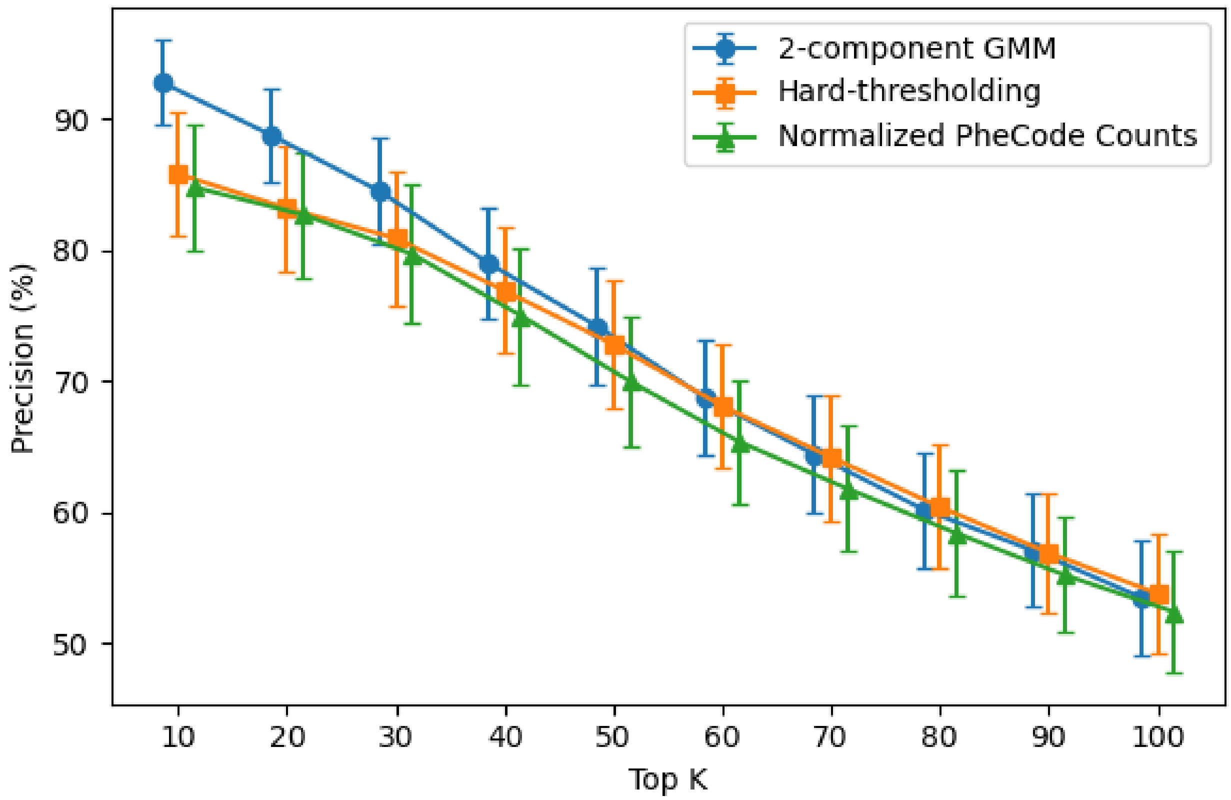

Figure S6: **Comparison of phenotype-prior initialization strategies for incidence prediction.** We compare three initialization methods: (1) a 2-component GMM on PheCode counts, (2) hard-thresholding with a cutoff of 1, and (3) normalized PheCode counts. Performance is evaluated using Precision@K (%) on incidence prediction across the top 50 PheCode topics, at a 40% subsampling rate. Error bars denote standard deviations across runs.

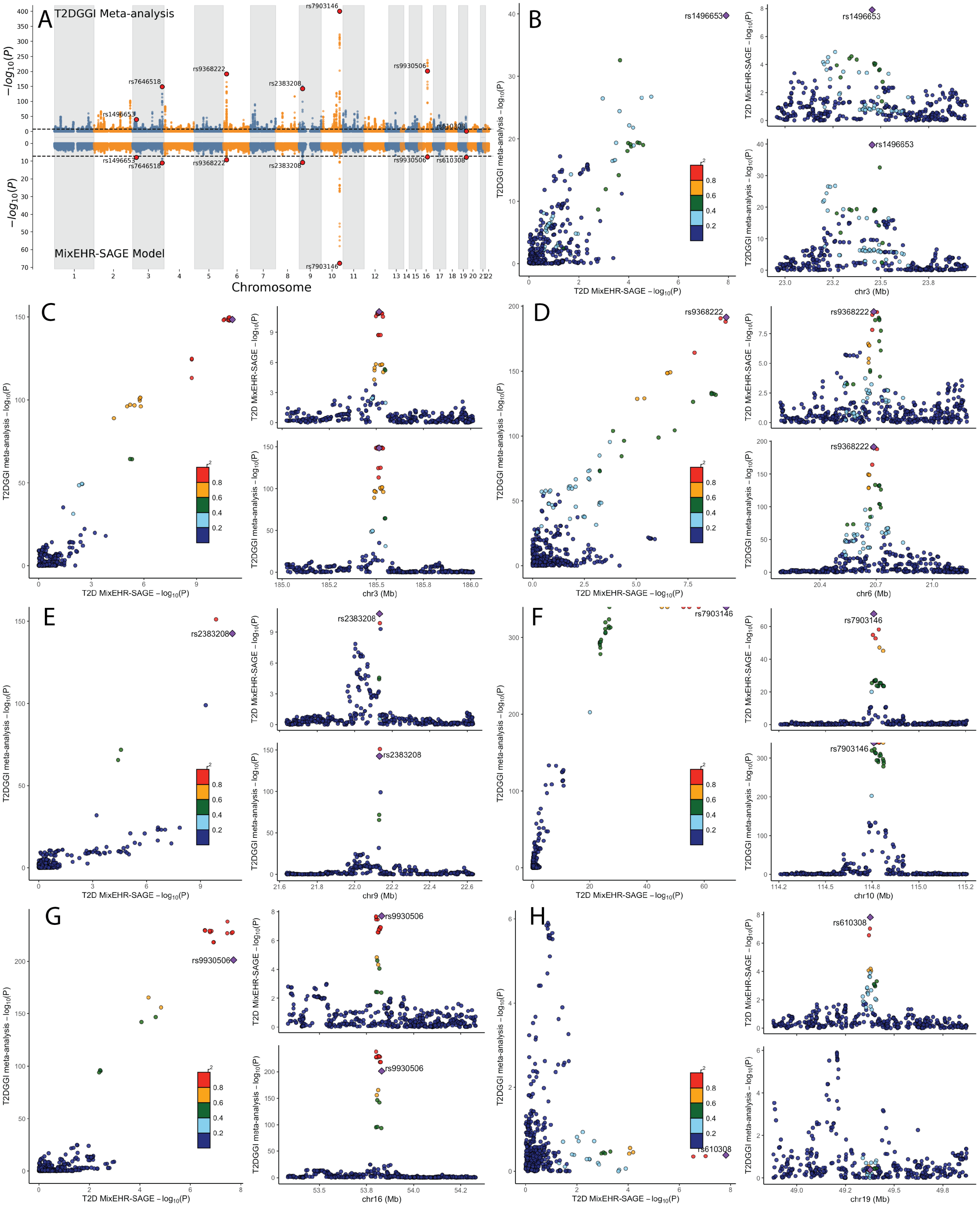

Figure S7: **Genome-wide and locus-specific comparison of T2D associations between the T2DGGI meta-analysis and the MixEHR-SAGE model** (A) Miami plot displaying GWAS comparison between T2DGGI meta-analysis and MixEHR-SAGE. Genome-wide significant loci were highlighted with their lead SNPs in red. (B–H) Regional plots for the genome-wide significant loci highlighted in panel A. For each subpanel, the left shows the locuscompare scatter plots of $-\log_{10}(P)$ values between the two studies with LD coloring (r^2 from 1000 Genomes EUR reference panel). The right subpanel shows regional association plots centered on the lead SNP.
